# Treatment‐related adverse events of antibody‐drug conjugates in clinical trials: A systematic review and meta‐analysis

**DOI:** 10.1002/cai2.97

**Published:** 2023-10-15

**Authors:** Jinming Li, Guoshuang Shen, Zhen Liu, Yaobang Liu, Miaozhou Wang, Fuxing Zhao, Dengfeng Ren, Qiqi Xie, Zitao Li, Zhilin Liu, Yi Zhao, Fei Ma, Xinlan Liu, Zhengbo Xu, Jiuda Zhao

**Affiliations:** ^1^ Breast Disease Diagnosis and Treatment Center Affiliated Hospital of Qinghai University, Affiliated Cancer Hospital of Qinghai University Xining Qinghai China; ^2^ Qinghai Provincial Clinical Research Center for Cancer; Qinghai Provincial Institute of Cancer Research Xining Qinghai China; ^3^ Department of Surgical Oncology General Hospital of Ningxia Medical University Yinchuan Ningxia Hui Autonomous Region China; ^4^ Qinghai University Xining Qinghai China

**Keywords:** adverse event, antibody‐drug conjugate, cancer, clinical trial, meta‐analysis

## Abstract

**Background:**

The wide use of antibody‐drug conjugates (ADCs) is transforming the cancer‐treatment landscape. Understanding the treatment‐related adverse events (AEs) of ADCs is crucial for their clinical application. We conducted a meta‐analysis to analyze the profile and incidence of AEs related to ADC use in the treatment of solid tumors and hematological malignancies.

**Methods:**

We searched the PubMed, Embase, and Cochrane Library databases for articles published from January 2001 to October 2022. The overall profile and incidence of all‐grade and grade ≥ 3 treatment‐related AEs were the primary outcomes of the analysis.

**Results:**

A total of 138 trials involving 15,473 patients were included in this study. The overall incidence of any‐grade treatment‐related AEs was 100.0% (95% confidence interval [CI]: 99.9%–100.0%; *I* ^2^ = 89%) and the incidence of grade ≥ 3 treatment‐related AEs was 6.2% (95% CI: 3.0%–12.4%; *I*² = 99%).

**Conclusions:**

This study provides a comprehensive overview of AEs related to ADCs used for cancer treatment. ADC use resulted in a high incidence of any‐grade AEs but a low incidence of grade ≥ 3 AEs. The AE profiles and incidence differed according to cancer type, ADC type, and ADC components.

## Graphical abstract

The wide use of antibody‐drug conjugates is transforming the cancer treatment landscape. Understanding the treatment‐related adverse events of antibody–drug conjugates is crucial for clinical application.

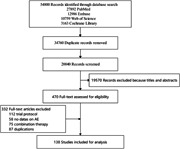

**Abbreviations**
ADCs: antibody‐drug conjugates
AE: adverse event
CI: confidence interval
RCTs: randomized controlled trials

## 1 INTRODUCTION

The emergence of antibody‐drug conjugates (ADCs) implied a paradigm shift in the treatment of many solid and hematologic malignancies. To date, 14 ADCs have been approved by the US Food and Drug Administration, the European Medicines Agency, National Medical Products Administration in China, and Japan's Ministry of Health, Labour and Welfare, for the treatment of solid tumors and hematological malignancies (Supporting Information: eTable 1) [1, 2, 3, 4, 5, 6, 7].

In addition, over 100 ADCs are currently being evaluated in clinical trials worldwide [2]. ADCs are highly potent biopharmaceutical drugs linking a cytotoxic agent (payload) to a monoclonal antibody via a chemical linker, thus allowing the preferential delivery of toxic payloads to cancer cells while sparing normal cells [1, 2, 3].

The wide use of ADCs requires a complete understanding of their toxicologic profile to allow the selection of a safe and efficacious dose. Limitations such as target specificity, linker stability, payload delivery, and the payload itself can induce adverse effects, which can be specific or even life‐threatening [8, 9, 10]. For instance, several randomized controlled trials (RCTs) demonstrated that ADCs can cause interstitial lung disease, ocular toxicity, serious organ dysfunction, anaphylaxis, severe thrombocytopenia, and neutropenia, as well as gastrointestinal effects [8, 11]. These adverse events (AEs) remain a significant challenge to the effective clinical application of ADCs. Despite this evidence, there has been no exhaustive overview of the profiles, incidence, and features of ADC‐related AEs. There is thus a need to summarize the profiles and incidence of ADC‐related AEs using standardized methods, to assist clinicians and researchers to manage these AEs and optimize the trial design of ADCs.

We conducted a systematic review and meta‐analysis of published clinical trials reporting treatment‐related AEs of ADCs approved by drug administrations worldwide, to provide complete profiles and data on the incidence of ADC‐related AEs. We also quantified potential differences in the incidence of AEs across various cancer types, ADC drugs, and ADC components.

## 2 EVIDENCE ACQUISITION

### 2.1 Search strategy and selection criteria

This systematic review and meta‐analysis was conducted according to the Preferred Reporting Items for Systematic Reviews and Meta‐Analysis guidelines [12]. We performed a systematic search of the literature to identify published clinical trials of ADCs that reported treatment‐related AEs. We searched the terms “antibody‐drug conjugates,” “cancer,” and “clinical trials” in PubMed, Embase, and the Cochrane Library to identify relevant studies published in English between January 2001 and October 2022 (Supporting Information: eTable 2). We also searched the reference lists of relevant review articles manually to identify additional eligible studies.

The inclusion criteria were as follows: (1) prospective clinical trial for cancer treatment between January 2001 and October 2022; (2) treatment with single‐agent ADC; (3) ADC drugs approved by any governmental drug administration worldwide; (4) reported overall incidence or tabulated data on treatment‐related AEs; and (5) published in English. We excluded conference abstracts that did not contain detailed AE‐related data and that had <10 trial participants. The literature search, study selection, and data extraction were performed independently by two authors (G.S. and J.L.). Discrepancies were resolved by discussion with a third reviewer (Z.L.) until consensus was achieved. If multiple articles described the same trial, the article with the most recent and/or comprehensive AE data was used.

### 2.2 Data analysis

The trial name, first author, year of publication, region, ADC used, ADC component (antibody, linker, and payload), trial phase, cancer type, study design, total number of participants, number of participants in safety analysis, arms and treatment, Common Terminology Criteria for Adverse Events (CTCAE) version, follow‐up time, and total number of all‐grade AEs were extracted for each study. The definitions of AEs were based on the Medical Dictionary for Regulatory Activities. Any‐grade, grade 3 or 4 AEs, and treatment‐related deaths were defined according to the CTCAE; treatment‐related AEs associated with treatment discontinuation were also extracted.

### 2.3 Outcomes

The primary outcome of interest was the overall incidence and profile of ADC‐related any‐grade and grade ≥ 3 AEs. The overall incidence and profile were determined by dividing the number of patients with all‐grade AEs by the total number of patients and the incidence of each specific AE, respectively. The incidence and profile of some specific AEs likely related to ADCs, subgroups according to cancer types, and the type of ADC drugs were analyzed. We also conducted subgroup analysis based on ADC components.

### 2.4 Statistical analysis

The effect size in our study was the incidence of AEs, obtained by dividing the number of participants with AEs by the total number of participants. Because the AE incidence did not follow a normal distribution, we performed logit conversion for AE incidence before the meta‐analysis [13, 14]. The pooled incidence with 95% confidence intervals (CIs) was calculated using generalized linear mixed models (GLMMs) [15]. When the number of AEs and total number of people included in the study were identical in all arms, the GLMM could not be fitted and a random‐effects model with restricted maximum likelihood estimation was used. Subgroup analyses of AE incidence were performed according to ADC drug, linker, payload, target, and tumor type. Heterogeneity between arms was assessed using the *χ* ^2^ test and *I* ^2^ statistic [16]. Heterogeneity was calculated using the Cochrane Q statistic and the *I* ^2^ test. Statistical heterogeneity was defined as *p* < 0.1 and/or *I* ^2^ > 50%. Publication bias was evaluated using a modified funnel plot of log odds against the sample size, because a conventional funnel plot against the standard error might be unreliable when the incidence is close to 0% or 100% [17]. Egger's test using sample size as a predictor was used to investigate publication bias [18]. The metafor and forestplot packages in R v.4.0.4 (www.r-project.org) were used for meta‐analysis and forest plot construction, respectively. *p* < 0.05 was considered statistically significant.

## 3 EVIDENCE SYNTHESIS

**Figure 1:**
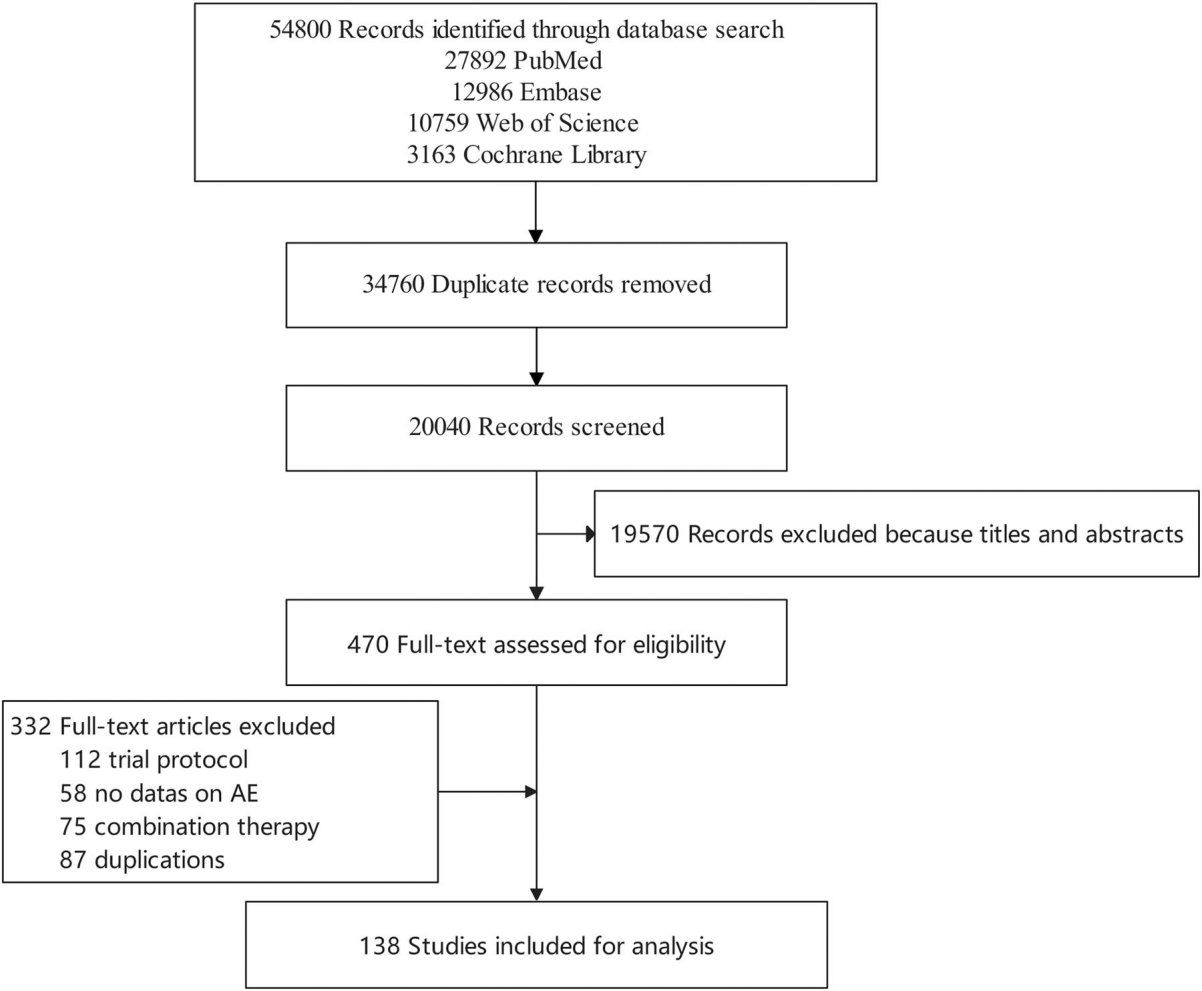
Flowchart of the search strategy.

Figure 1 presents a flowchart of the search strategy. The electronic searches yielded 54,800 potentially relevant publications, of which 470 studies were potentially eligible. After screening based on the eligibility criteria, 138 studies (108 RCTs and 30 cohort studies) involving 15,473 participants were included [19, 20, 21, 22, 23, 24, 25, 26, 27, 28, 29, 30, 31, 32, 33, 34, 35, 36, 37, 38, 39, 40, 41, 42, 43, 44, 45, 46, 47, 48, 49, 50, 51, 52, 53, 54, 55, 56, 57, 58, 59, 60, 61, 62, 63, 64, 65, 66, 67, 68, 69, 70, 71, 72, 73, 74, 75, 76, 77, 78, 79, 80, 81, 82, 83, 84, 85, 86, 87, 88, 89, 90, 91, 92, 93, 94, 95, 96, 97, 98, 99, 100, 101, 102, 103, 104, 105, 106, 107, 108, 109, 110, 111, 112, 113, 114, 115, 116, 117, 118, 119, 120, 121, 122, 123, 124, 125, 126, 127, 128, 129, 130, 131, 132, 133, 134, 135, 136, 137, 138, 139, 140, 141, 142, 143, 144, 145, 146, 147, 148, 149, 150, 151, 152, 153, 154, 155, 156]. The ADCs used included gemtuzumab ozogamicin (*n* = 12), brentuximab vedotin (*n* = 32), trastuzumab emtansine (*n* = 17), inotuzumab ozogamicin (*n* = 11), moxetumomab pasudotox (*n* = 7), polatuzumab vedotin‐piiq (*n* = 1), enfortumab vedotin (*n* = 7), trastuzumab deruxtecan (*n* = 15), sacituzumab govitecan (*n* = 11), belantamab mafodotin‐blmf (*n* = 6), loncastuximab tesirine‐lpyl (*n* = 5), tisotumab vedotin‐tftv (*n* = 5), disitamab vedotin (*n* = 8), and RM‐1929 (*n* = 1). The basic characteristics of the included studies are shown in Table 1. For all studies, ADC therapy was evaluated in a relapsed, refractory, advanced, or metastatic setting, and most studies reported AEs as secondary outcomes. The cancer types included breast cancer (*n* = 28), cervical cancer (*n* = 5), gastric cancer or other solid tumors (*n* = 10), urothelial carcinoma (*n* = 12), lung cancer (*n* = 6), head and neck squamous cell carcinoma (*n* = 2), lymphoma (*n* = 34), leukemia (*n* = 35), and multiple myeloma (*n* = 6). In total, 138 trials (15,473 patients) and 110 trials (14,183 patients) were included for analyses of the profile and overall incidence of ADC‐related AEs, respectively.

**Table 1:** Main characteristics of the included clinical studies.

First author, year	Study	Cancer	Journal	Country	Trial phase	Median follow‐up time, months	Total number	Safety analysis number	Arm	Treatment	Adverse events study	CTCAE version
Zhi Peng, 2021	Zhi Peng, 2021	Gastric or gastroesophageal junction cancer	*Cancer Communications*	China	Phase II	12 m	125	118	Single	RC48	Secondary outcomes	4.03
Xinan Sheng, 2020	Xinan Sheng, 2020	Urothelial carcinoma	*Clin Cancer Res*	China	Phase II	20.3 m	43	43	Single	RC48	Secondary outcomes	
Yingying Xu, 2021	Yingying Xu, 2021	Advanced solid tumors	*Gastric Cancer*	China	Phase I	NS	57	46	Single	RC48 2.0 mg/kg Q2W	Secondary outcomes	4
Jiayu Wang, 2019	C001 Cancer C003 Cancer	Breast cancer	*ASCO*	China	Phase I	NS	118	118	Single	mg/kg	Primary endpoint	
Jifang Gong, 2022	NCT02881190	Gastric cancer or other solid tumors	*ASCO*	China	Phase I	NS	36	36	Single	2.5 mg/kg Q2W	Primary endpoint	
Xinan Sheng, 2022	RC48‐C005 RC48‐C009	Urothelial carcinoma	*ASCO*	China	Phase II	NS	107	107	Single	RC48	Secondary outcomes	
Huayan Xu, 2022	Huayan Xu, 2022	Urothelial carcinoma	*ASCO*	China	Phase II	NS	19	19	Single	2 mg/kg Q2W	Primary endpoint	
Xinan Sheng, 2021	RC48‐C009	Urothelial carcinoma	*ASCO*	China	Phase II	NS	64	64	Single	2 mg/kg Q2W	Secondary outcomes	
Javier Cortés, 2022	DESTINY‐Breast03	Breast cancer	*N Engl J Med*	USA	Phase III	16.2 m	261	257	Double	5.4 mg/kg Q3W	Secondary outcomes	5
Salvatore Siena, 2022	DESTINY‐CRC01	Colorectal cancer	*Lancet Oncol*	Japan	Phase II	27.1 m	78	78	Single	6.4 mg/kg Q3W	Secondary outcomes	4.03/5.0
Kenji Tamura, 2022	JapicCTI‐152978	Breast cancer	*Lancet Oncol*	Japan	Phase I	9.9 m	118	115	Single	5.4 mg/kg or 6.4 mg/kg Q3W	Primary endpoint	4
Kohei Shitara, 2022	JapicCTI‐152978	Gastric cancer	*Lancet Oncol*	Japan	Phase I	5.5 m	44	44	Single	5.4 mg/kg or 6.4 mg/kg Q3W	Primary endpoint	4
Bob T. Li, 2021	DESTINY‐Lung01	Non‐small cell lung cancer	*N Engl J Med*	USA	Phase II	13.1 m	91	91	Single	6.4 mg/kg Q3W	Secondary outcomes	5
S. Modi, 2022	DESTINY‐Breast01	Breast cancer	*N Engl J Med*	USA	Phase II	11 m	184	184	Single	5.4 mg/kg Q3W	Secondary outcomes	4.03
Junji Tsurutani, 2020	Junji Tsurutani, 2020	Non‐small cell lung cancer or colorectal cancer or other solid cancer	*Cancer Discov*	Japan	Phase I	7.8 m	60	59	Single	6.4 mg/kg Q3W	Secondary outcomes	4.03
Kohei Shitara, 2022	DESTINY‐Gastric01	Gastric cancer	*N Engl J Med*	Japan	Phase II	NS	125	125	Double	5.4 mg/kg (breast cancer) or 6.4 mg/kg Q3W	Secondary outcomes	4.03
S. Modi, 2022	DESTINY‐Breast04	Breast cancer	*N Engl J Med*		Phase III	18.4 m	373	371	Double	5.4 mg/kg Q3W	Secondary outcomes	5
Shanu Modi, 2020	Shanu Modi, 2020	Breast cancer	*J Thorac Oncol*	USA	Phase I	NS	54	54	Single	5.4 or 6.4 mg/kg Q3W	Primary endpoint	4.0
Véronique Diéras, 2022	Daisy	Breast cancer	*Cancer Research*		Phase II	10.1 m	179	179	Single	5.4 mg/kg Q3W	Secondary outcomes	
Hideaki Bando, 2021	FIH, DDI	Salivary duct carcinoma	*ASCO*		Phase I	NS	17	17	Single	6.4 mg/kg Q3W 5.4 mg/kg Q3W	Secondary outcomes	
Toshinari Yamashita, 2020	Toshinari Yamashita, 2020	Breast cancer	*Cancer Res*		Phase I	4.8 m	51	51	Single	6.4 mg/kg Q3W	Secondary outcomes	
R. Bartsch, 2022	TUXEDO‐1	Breast cancer	*Ann Oncol*		Phase II	11 m	15	15	Single	_	Secondary outcomes	
Akihiro Ohba, 2022	HERB	Biliary tract cancer	*ASCO*	Japan	Phase II	NS	32	32	Single	5.4 mg/kg Q3W	Secondary outcomes	
Muralidhar Beeram, 2014	TDM3569g	Breast cancer	*Cancer*	USA	Phase I	NS	28	28	Single	2.0 mg/kg 2.4 mg/kg 2.9 mg/kg QW Q3W	Primary endpoint	3
Bob T. Li, 2018	Bob T. Li, 2018	Lung adenocarcinomas	*J Clin Oncol*	USA	Phase II	10 m	18	18	Single	3.6 mg/kg Q3W	Secondary outcomes	4.1
Katsuyuki Hotta, 2017	Katsuyuki Hotta, 2017	Non‐small cell lung cancer	*J Thorac Oncol*	Japan	Phase II	9.2 m	15	15	Single	3.6 mg/kg Q3W	Secondary outcomes	
Sara M. Tolaney, 2019	ATEMPT	Breast cancer	*J Clin Oncol*	USA	Phase II	45 m	383	383	Double	3.6 mg/kg Q3W	Primary endpoint	
Sara A. Hurvitz, 2020	TDM4450g	Breast cancer	*J Clin Oncol*	USA	Phase II	23 m	69	69	Double	3.6 mg/kg Q3W	Primary endpoint	3
Harukaze Yamamoto, 2015	Harukaze Yamamoto, 2015	Breast cancer	*Jap J Clin Oncol*	Japan	Phase I	NS	10	10	Single	1.8,2.4 or 3.6 mg/kg Q3W	Secondary outcomes	3
Ian E. Krop, 2010	Ian E. Krop, 2010	Breast cancer	*J Clin Oncol*	USA	Phase I	NS	24	24	Single	0.3, 0.6, 1.2, 2.4, 3.6, 4.8 mg/kg Q3W	Primary endpoint	3
Howard A. Burris III, 2022	TDM4258g	Breast cancer	*J Clin Oncol*	USA	Phase II	12 m	112	112	Single	3.6 mg/kg Q3W	Primary endpoint	
Filippo Montemurro, 2021	KAMILLA	Breast cancer	*Eur J Cancer*	Italy	Phase III	20.6 m	2002	2002	Single	3.6 mg/kg Q3W	Primary endpoint	4.04
Sunil Verma, 2022	EMILIA	Breast cancer	*N Engl J Med*	Canada	Phase III	13 m	495	490	Double	2.4 3.0 3.6 mg/kg Q3W	Primary endpoint	3
Solange Peters, 2018	Solange Peters, 2018	Non‐small cell lung cancer	*Am Assoc Cancer Res*	Switzerland	Phase II	23.1 m	49	49	Single	3.6 mg/kg Q3W	Secondary outcomes	4
Eiji Iwama, 2022	JapicCTI‐194620	Non‐small cell lung cancer	*Eur J Cancer*	Japan	Phase II	8 m	22	22	Single	3.6 mg/kg Q3W	Secondary outcomes	4.1
G. von Minckwitz, 2022	Katherine	Breast cancer	*N Engl J Med*	USA	Phase III	40.9 m	743	740	Double	3.6 mg/kg Q3W	Secondary outcomes	
Ian E. Krop, 2017	TH3RESA	Breast cancer	*Lancet Oncol*	USA	Phase III	7.2 m	404	403	Double	3.6 mg/kg Q3W	Secondary outcomes	4
Javier Cortés, 2021	DESTINY‐Breast03	Breast cancer	*N Engl J Med*	USA	Phase III	6.9 m	263	261	Double	3.6 mg/kg Q3W	Secondary outcomes	5
	Gatsby	Gastric or gastro‐esophageal junction adenocarcinoma	*Lancet Oncol*	Korea	Phase II/III	17.5 m	228	224	Double	2.4 mg/kg QW	Secondary outcomes	4.03
Edith A. Perez, 2018	Marianne	Breast cancer	*Cancer*	USA	Phase III	35 m	367	361	Triple	3.6 mg/kg Q3W	Secondary outcomes	
Ian E. Krop, 2017	TDM4874g	Breast cancer	*J Clin Oncol*	USA	Phase II	24.6 m	148	148	Single	3.6 mg/kg Q3W	Secondary outcomes	4
Erica Brivio, 2017	ITCC‐059	Acute lymphocytic leukemia	*Blood*	Netherland	Phase I	19 m	25	25	Single	1.4, 1.8 mg/kg QW	Primary outcome	
Andre Goy, 2021	Andre Goy	Follicular lymphoma	*Br J Hematol*	USA	Phase II	24 m	72	72	Single	1.8 mg/kg Q4W	Secondary outcomes	
Edoardo Pennesi, 2022	ITCC‐059	Acute lymphocytic leukemia	*Leukemia*	Netherland	Phase II	16 m	28	28	Single	1.8 mg/kg Q3W	Secondary outcomes	
Hagop Kantarjian, 2012	Hagop Kantarjian, 2012	Acute lymphocytic leukemia	*Lancet Oncol*	USA	Phase II	NS	49	49	Single	1.8 mg/kg Q3‐4W	Secondary outcomes	3
Hagop M. Kantarjian, 2016	Hagop M. Kantarjian, 2016	Acute lymphocytic leukemia	*N Engl J Med*	USA	Phase III	NS	139	139	Double	1.8, 0.8, 0.5 mg/m^2^ Q3‐4W	Secondary outcomes	
Daniel J. DeAngelo, 2017	Daniel J. DeAngelo, 2017	Acute lymphocytic leukemia	*Blood Adv*	USA	Phase I	23.7 m	60	60	Single	1.6 mg/m^2^ D1, 8, 15 Q4W	Secondary outcomes	3
Michinori Ogura, 2010	Michinori Ogura, 2010	Follicular lymphoma	*Cancer Sci*	Japan	Phase I	NS	10	10	Single	1.8 mg ⁄ m^2^ Q4W	Primary outcome	3
Hagop Kantarjian, 2013	Hagop Kantarjian, 2013	Acute lymphocytic leukemia	*Cancer*	USA	Phase II	21 m	90	90	Single	Weekly 0.8 mg/m^2^ D1, 0.5 mg/m^2^, D8, D15 Q3‐4W		
Hagop M. Kantarjian, 2019	INO‐VATE	Acute lymphocytic leukemia	*Cancer*	USA	Phase III	24 m	164	164	Double	1.8 mg/kg Q3‐4W	Secondary outcomes	3
Hagop M. Kantarjian, 2020	INO‐VATE	Acute lymphocytic leukemia	*Lancet Haematol*	USA	Phase III	NS	164	164	Double	1.8 mg/kg Q3‐4W	Secondary outcomes	3
Daniel J. DeAngelo, 2020	INO‐VATE	Acute lymphocytic leukemia	*Blood Cancer J*	USA	Phase III	NS	162	162	Double	1.8 mg/kg Q3‐4W	Secondary outcomes	3
Johann S. de Bono, 2019	InnovaTV 201	Advanced or metastatic solid tumors	*Lancet*	UK	Phase I/II	2.8 m	147	147	Single	2.0 mg/kg Q3W	Primary outcome	4.03
David S. Hong, 2019	InnovaTV 201	Recurrent or metastatic cervical cancer	*Clin Cancer Res*	USA	Phase I/II	3.5 m	55		Single	2.0 mg/kg Q3W	Primary outcome	4.03
Robert L. Coleman, 2021	InnovaTV204/GOG‐3023/ENGOT‐cx6	Recurrent or metastatic cervical cancer	*Lancet*	USA	Phase II	10.0 m	101	101	Single	2.0 mg/kg Q3W	Primary outcome	5.0
Kan Yonemori, 2022	InnovaTV206	Cervical cancer	*Cancer Sci*	Japan	Phase I/II	14.0 m	23	17	Single	2.0 mg/kg Q3W	Primary outcome	5.0
D.S. Hong, 2022	D.S. Hong, 2022	Head and neck squamous cell carcinoma	*Int J Radiat Oncol*	USA	Phase I/II	NS	31	31	Single	2.0 mg/kg Q3W	Secondary outcomes	
Aditya Bardia, 2017	Aditya Bardia, 2017	Triple‐negative breast cancer	*J Clin Oncol*	USA	Phase I/II	16.6 m	69		Single	10 mg/kg Q3W	Primary outcome	4.03
A. Bardia, 2019	IMMU‐132‐01	Triple‐negative breast cancer	*N Engl J Med*	USA	Phase I/II	NS	108		Single	10 mg/kg Q3W	Primary outcome	4.0
A. Bardia, 2021	IMMU‐132‐01	Epithelial cancer	*Ann Oncol*	USA	Phase I/II	8.97 m	495	495	Single	8, 10, 12, or 18 mg/kg Q3W	Primary outcome?	4.3
A. Bardia, 2021	ASCENT	Triple0negative breast cancer	*N Engl J Med*	USA	Phase III	17.7 m	258	258	Double	10 mg/kg Q3W	Secondary outcomes	4.03
Lisa A. Carey, 2022	ASCENT	Breast cancer	*BPJ*	USA	Phase III	NS	33	33	Double	10 mg/kg Q3W	Secondary outcomes	4.03
Lisa A. Carey, 2022	ASCENT	Breast cancer	*AACR*	USA	Phase III	NS	28	25	Double	10 mg/kg Q3W	Secondary outcomes	
Laura Spring, 2022	NeoSTAR	Triple‐negative breast cancer	*ASCO*	USA	Phase II	NS	50	50	Single	10 mg/kg Q3W	Secondary outcomes	
F. Marmé, 2022	SASCIA	Breast cancer	*Ann Oncol*	Germany	Phase III	28 m	45	45	Double	_	Primary outcome	
Alessandro Santin, 2020	Alessandro Santin, 2020	Endometrial cancer	*ASCO*	USA	Phase I/II	NS	495	495	Single	10 mg/kg Q3W	Primary outcome	
K. Kalinsky, 2020	K. Kalinsky, 2020	Breast cancer	*Ann Oncol*	USA	Phase I/II	11.5 m	54	54	Single	10 mg/kg Q3W	Secondary outcomes	4.3
Scott T. Tagawa, 2021	TROPHY‐U‐01	Urothelial carcinoma	*J Clin Oncol*	USA	Phase II	9.1 m	113	113	Single	10 mg/kg Q3W	Secondary outcomes	5.0
David M. Cognetti MD, 2021	David M. Cognetti MD, 2021	Head and neck squamous cell carcinoma	*Head & Neck*	USA	Phase I/IIa	NS	30		Single	640 mg/m^2^ with fixed light dose (50 J/cm^2^ or 100 J/cm)	Primary outcome	4.03
Maria Corinna A. Palanca‐Wessels, 2015	Maria Corinna A. Palanca‐Wessels, 2015	Non‐Hodgkin lymphoma	*Lancet*	USA	Phase I	4.3 m	75	75	Single	＜1.8 mg/kg Q3W	Primary outcome	4.0
Shunji Takahashi, 2019	Shunji Takahashi, 2019	Locally advanced or metastatic urothelial carcinoma	*Investig N Drugs*	Japan	Phase I	NS	17		Double	1.0 mg/kg Q4W	Primary outcome	
Evan Y Yu, 2021	EV‐201	Advanced urothelial carcinoma	*Lancet*	USA	Phase II	13.4 m	89	89	Single	1.25 mg/kg Q4W	Secondary outcomes	4.03
Thomas Powles, 2021	EV‐301	Advanced urothelial carcinoma	*N Engl J Med*	USA	Phase III	11.1 m	301	296	Double	1.25 mg/kg Q4W	Secondary outcomes	4.03
Jonathan Rosenberg, 2020	EV‐101	Metastatic urothelial carcinoma	*J Clin Oncol*	USA	Phase I	16.4 m	153		Single	0.75 mg/kg Q4W	Primary outcome	4.03
Jonathan E. Rosenberg, 2019	EV‐201	Locally advanced or metastatic urothelial carcinoma	*J Clin Oncol*	USA	Phase II	10.2 m	125	125	Single	1.25 mg/kg Q4W	Secondary outcomes	4.03
Terence W. Friedlander, 2021	EV‐103	Locally advanced or metastatic urothelial carcinoma	*ASCO*	USA		24.9 m	45			EV+P (EV1.25 mg/kg Q4W+P day1)	Primary outcome	
Bradley Alexander McGregor, 2022	EV‐201	Locally advanced or metastatic urothelial carcinoma	*ASCO*	USA	Phase II		89	89	Single	1.25 mg/kg Q4W		
Robert J. Kreitman, 2018	Robert J. Kreitman, 2018	Hairy cell leukemia	*Leukemia*	USA		16.7 m	80	80	Single	40 µg/kg Q4W	Secondary outcomes	4.03
Nicholas J. Short, 2018	Nicholas J. Short, 2018	Acute lymphocytic leukemia	*Br J Hematol*	USA	Phase I	NS	16	16	Single	30 μg/kg or 40 μg/kg or 50 μg/kg Q3W	Primary outcome	
Robert J. Kreitman, 2018	Robert J. Kreitman, 2018	Hairy cell leukemia	*Blood*	USA	Phase I	NS	33		Single	50 µg/kg Q4W		
Robert J. Kreitman, 2021	Robert J. Kreitman, 2021	Hairy cell leukemia	*J Hematol Oncol*	USA		24.6 m	80	80	Single	40 µg/kg Q4W	Secondary outcomes	4.03
Alan S. Wayne, 2017	Alan S. Wayne, 2017	Acute lymphocytic leukemia	*Blood*	USA	Phase I	NS	55	55	Single	5–50 μg/kg Q3W	Primary outcome	
Robert J. Kreitman, 2012	Robert J. Kreitman, 2012	Hairy cell leukemia	*J Clin Oncol*	USA	Phase I	NS	28		Single	5–50 μg/kg Q3W	Primary outcome	3.0
Nirali N. Shah, 2020	Nirali N. Shah, 2020	Acute lymphocytic leukemia	*Pediatr Blood Cancer*	USA	Phase II	NS	30	30	Single	40 µg/kg Q3W	Secondary outcomes	
Suzanne Trudel, 2019	Suzanne Trudel, 2019	Multiple myeloma	*Blood Cancer J*	USA	Phase I	12.5 m	35	35	Single	3.4 mg/kg Q3W	Primary outcome	
Sagar Lonial, 2019	DREAMM‐2	Multiple myeloma	*Lancet*	USA	Phase II	6.3 m	194	194	Double	2.5 mg/kg Q3W	Secondary outcomes	4.03
Kyriaki Tzogani, 2021	Kyriaki Tzogani, 2021	Multiple myeloma	*Oncologist*	Netherlands	Phase II	NS	97		Double	2.5 mg/kg Q3W	Secondary outcomes	
Sagar Lonial, 2021	DREAMM‐2	Multiple myeloma	*Cancer*	USA	Phase II	NS	95	95	Double	2.5 mg/kg Q3W	Secondary outcomes	4.03
Paul G. Richardson, 2020	DREAMM‐2	Relapsed or refractory multiple myeloma	*Blood Cancer J*	USA	Phase II	11.2 m	24		Double	3.4 mg/kg Q3W N = 24	Secondary outcomes	4.03
Suzanne Trudel, 2018	BMA117159	Relapsed or refractory multiple myeloma	*Lancet*	Canada	Phase I	6.6 m	35	35	Single	3.4 mg/kg Q3W	Primary outcome	4.0
Brad S. Kahl, 2019	Brad S. Kahl, 2019	Non‐Hodgkin lymphoma	*Clin Cancer Res*	USA	Phase I	7.5 m	88	88	Single	15–200 µg/kg Q3W	Primary outcome	4.0
Nitin Jain, 2020	Nitin Jain, 2020	Acute lymphocytic leukemia	*Blood Adv*	USA	Phase I	NS	35	35	Single	15–150 µg/kg Q3W	Primary outcome	4.0
Paolo F Caimi, 2021	LOTIS‐2	Diffuse large B‐cell lymphoma	*Lancet*	USA	Phase II	NS	145	145	Single	150 µg/kg Q3W	Secondary outcomes	4.0
Paolo F Caimi, 2020	Paolo F Caimi, 2020	Diffuse large B‐cell lymphoma	*Blood*	USA	Phase II	NS	145	145		150 µg/kg Q3W	Secondary outcomes	4.0
Mehdi Hamadani, 2021	Mehdi Hamadani, 2021	Non‐Hodgkin lymphoma	*Blood*	USA	Phase I	NS	183	183	Single	15–200 µg/kg Q3W	Primary outcome	4.0
Pier Paolo Piccaluga, 2004	Pier Paolo Piccaluga, 2004	Acute myeloid leukemia	*Leukemia Lymphoma*	USA		NS	24		Single	1.5, 6, 9 mg/sqm Q2, 4 W	Primary outcome	
Francesco Lo‐Coco, 2016	Francesco Lo‐Coco, 2016	Acute promyelocyticleukemia	*Blood*	Italy		NS	16		Single	6 mg/m^2^ Q2W	Secondary outcomes	
S. Amadori, 2005	S. Amadori, 2005	Acute myeloid leukemia	*Leukemia*	Italy	Phase II	NS	40		Single	69 mg/m^2^ Q2W	Secondary outcomes	
Sergio Amadori, 2016	AML‐19	Acute myeloid leukemia	*J Clin Oncol*	Italy	Phase III	NS	111	111	Double	GO	Secondary outcomes	3.0
A‐L Taksin, 2007	A‐L Taksin, 2007	Acute myeloid leukemia	*Leukemia*	France	Phase II	NS	57		Single	3 mg/m^2^/day on days 1, 4 and 7	Primary outcome	2.0
Yukio Kobayashi, 2009	Yukio Kobayashi, 2009	Acute myeloid leukemia	*Int J Hematol*	Japan	Phase I	NS	40		Single	6, 7.5, 9 mg/m^2^	Primary outcome	2.0
Chadi Nabhan, 2005	Chadi Nabhan, 2005	Acute myeloid leukemia	*Leukemia Res*	USA	Phase II	NS	12		Single	9 mg/m^2^ Q2W	Secondary outcomes	
Robert J. Arceci, 2016	Robert J. Arceci, 2016	Acute myeloid leukemia	*Blood*	USA	Phase II	NS	29		Single	6–9 mg/m^2^ Q2W	Primary outcome	1.0
Christian M. Zwaan, 2009	AML 2001/02	Acute myeloid leukemia	*Br J Hematol*	Netherlands	Phase II	>36 m	30		Single	7 ± 5 mg/m^2^ Q2W	Primary outcome	2.0
By Eric L. Sievers, 2001	By Eric L. Sievers, 2001	Acute myeloid leukemia	*J Clin Oncol*	USA	Phase II	NS	142		Single	9 mg/m^2^ Q2W		
Richard A. Larson, 2005	Richard A. Larson, 2005	Acute myeloid leukemia	*Cancer*	USA	Phase II	NS	277	277	Single	9 mg/m^2^ Q2W		
Eunice S. Wang, 2020	EAP study	Acute myeloid leukemia	*Leukemia Lymphoma*	USA		NS	139	139	Double	3–9 mg/m^2^ Q2W	Primary outcome	4.03
Jeffrey P. Sharman, 2019	Jeffrey P. Sharman, 2019	CD30‐expressing nonlymphomatous cancer	*Investig N Drugs*	USA	Phase II	NS	63	63	Single	1.8 or 2.4 mg/kg Q3W	Secondary outcomes	4.03
Jason Gotlib, 2019	Jason Gotlib, 2019	AdvSM	*Blood Adv*	USA	Phase II	23.8 m	10		Single	1.8 mg/kg Q3W	Secondary outcomes	4.03
Michelle A. Fanale, 2011	Michelle A. Fanale, 2011	Hodgkin lymphoma	*Clin Cancer Res*	USA	Phase I	4.5 m	44		Single	0.4–1.4 mg/kg Q4W	Primary outcome	3.0
Miso Kim, 2021	Miso Kim, 2021	Non‐Hodgkin lymphoma	*Haematologica*	Korea	Phase II	20 m	25		Single	1.8 mg/kg Q3W	Secondary outcomes	4.03
Barbara Pro, 2012	Barbara Pro, 2012	Acute lymphocytic leukemia	*J Clin Oncol*	USA	Phase II	NS	58		Single	1.8 mg/kg Q3W	Secondary outcomes	3.0
Ajay K. Gopal, 2012	Ajay K. Gopal, 2012	Hodgkin lymphoma	*Blood*	USA	Phase III	34w	25	25	Single	1.8, 1.2 mg/kg Q3W	Secondary outcomes	
Anas Younes, 2010	Anas Younes, 2010	Hodgkin lymphoma	*M Engl J Med*	USA	Phase I	NS	45			0.1–3.6 mg/kg Q3W	Primary outcome	3.0
Craig H Moskowitz, 2015	AETHERA	Hodgkin lymphoma	*Lancet*	USA	Phase III	NS	167	167	Double	1.8 mg/kg Q3W	Secondary outcomes	4.0
Eric D. Jacobsen, 2015	Eric D. Jacobsen, 2015	Diffuse large B‐cell lymphoma	*Blood*	USA	Phase II	4.6 m	49	49	Single	1.8 mg/kg Q3W	Secondary outcomes	4.03
Nancy L Bartlett, 2014	Nancy L Bartlett, 2014	Hodgkin lymphoma	*J Hematol Oncol*	USA	Phase II	NS	29	29	Single	1.8 mg/kg Q3W	Primary outcome	3
Franco Locatelli, 2018	Franco Locatelli, 2018	Hodgkin lymphoma	*Lancet*	Italy	Phase I/II	NS	36	36	Single	1.4 or 1.8 mg/kg Q3W	Secondary outcomes	4.03
Anas Younes, 2012	Anas Younes, 2012	Hodgkin lymphoma	*J Clin Oncol*	USA	Phase II	NS	102	102	Single	1.8 mg/kg Q3W	Secondary outcomes	3
Vittorio Stefoni, 2020	FIL_BVHD01	Hodgkin lymphoma	*Haematologica*	Italy	Phase II	24.9 m	20	18	Single	1.8 mg/kg Q3W	Secondary outcomes	
Andres Forero‐Torres, 2012	Andres Forero‐Torres, 2012	Hodgkin lymphoma	*Oncologist*	USA	Phase I	>18 m	20	20	Single	0.1–2.7 mg/kg Q3W or QW	Secondary outcomes	3.0
H Miles Prince, 2017	ALCANZA	Anaplastic large cell lymphoma	*Lancet*	Australia	Phase III	22.9 m	66	66	Double	1.8 mg/kg Q3W	Secondary outcomes	4.03
Baiteng Zhao, 2016	Baiteng Zhao, 2016	Hepatic impairment	*Br J Clin Pharmacol*	USA	Phase I	NS	7	7	Double	1.2 mg/kg 3 W	Secondary outcomes	3.0
Seok Jin Kim, 2020	Seok Jin Kim, 2020	Non‐Hodgkin lymphoma	*Cancer Res Treat*	Korea	Phase II	29.9 m	33	33	Single	1.8 mg/kg Q3W	Secondary outcomes	
Robert Chen, 2016	Robert Chen, 2016	Hodgkin lymphoma	*Blood*	USA	Phase II	NS	34		Single	1.8 mg/kg Q3W	Secondary outcomes	3.0
Craig H. Moskowitz, 2018	AETHERA	Hodgkin lymphoma	*Blood*	USA	Phase III	NS	165		Double	1.8 mg/kg Q3W	Secondary outcomes	
Alessandra Romano, 2019	Alessandra Romano, 2019	Hodgkin lymphoma	*Br J Hematol*	Italy		47.3 m	40		Single	1.8 mg/kg Q3W	Secondary outcomes	3.0
Philippe Armand, 2018	CheckMate 205	Hodgkin lymphoma	*J Clin Oncol*	USA	Phase II	18 m	243	243	Single	3 mg/kg Q2W	Secondary outcomes	
John Kuruvilla, 2021	KEYNOTE‐204	Hodgkin lymphoma	*Lancet*	Canada	Phase III	24 m	153	153	Double	1.8 mg/kg Q3W	Secondary outcomes	4.0
Jan Walewski, 2018	Jan Walewski, 2018	Hodgkin lymphoma	*Br J Hematol*	Poland	Phase Ⅳ	6.9 m & 16.6 m	60	60	Single	1.8 mg/kg Q3W	Secondary outcomes	4.03
Steven M. Horwitz, 2021	ALCANZA	Hodgkin lymphoma	*Blood Adv*	USA	Phase III	45.9 m	66	66	Double	1.8 mg/kg Q3W	Secondary outcomes	4.03
Youn H, 2015	Youn H, 2015	Mycosis fungoides (MF) and Sézary syndrome (SS)	*J Clin Oncol*	American	Phase II	NA	32	32	Single	1.8 mg/kg Q3W	Secondary outcomes	4.0
Zachariah DeFilipp, 2018	Zachariah DeFilipp, 2018	Steroid‐refractory chronic graft‐versus‐host disease (cGVHD)	*Biol Blood Marrow Transplant*	American	Phase I	35 m	17	17	Single	0.3–1.8 mg/kg Q3W	Secondary outcomes	
Andres Forero‐Torres, 2015	Andres Forero‐Torres, 2015	Hodgkin lymphoma	*Am Soc Hematol*	American	Phase II	29 m	27	27	Single	1.8 mg/kg Q3W	Secondary outcomes	
Michinori Ogura, 2014	Michinori Ogura, 2014	Hodgkin lymphoma	*Cancer Sci*	Japan	Phase I/II	19 m	20	20	Single	1.8 mg/kg Q3W	Secondary outcomes	
Robert Chen, 2015	Robert Chen, 2015	Hodgkin lymphoma	*Biol Blood Marrow Transplant*	USA	Phase II	NS	37	37	Single	1.8 mg/kg Q3W	Secondary outcomes	4.03
Madeleine Duvic, 2013	Madeleine Duvic, 2013	Hodgkin lymphoma	*Blood*	USA	Phase II	NS	48	48	Single	1.8 mg/kg Q3W	Primary outcome	
Ryan Ashkar, 2021	Ryan Ashkar, 2021	Giant cell tumor of bone	*Investig N Drugs*	USA	Phase II	NS	18		Single	1.8 mg/kg Q3W	Secondary outcomes	4.0
Yuqin Song, 2021	Yuqin Song, 2021	Hodgkin lymphoma	*Expert Rev Hematol*	China	Phase II	16.6 m	39	39	Single	1.8 mg/kg Q3W	Secondary outcomes	

### 3.1 Overall incidence of AEs

In general, these studies reported >300 types of AEs. A total of 14,320 (92%) among 15,473 participants from 138 studies experienced at least one any‐grade AE and 2,695 (20.57%) of 13,101 participants from 98 studies experienced at least one grade ≥ 3 AE. Considering that this study mainly focused on grade ≥ 3 and any‐grade AEs, and we restricted ADC‐related AEs to the 28 most common, important, or specific AEs.

**Figure 2:**
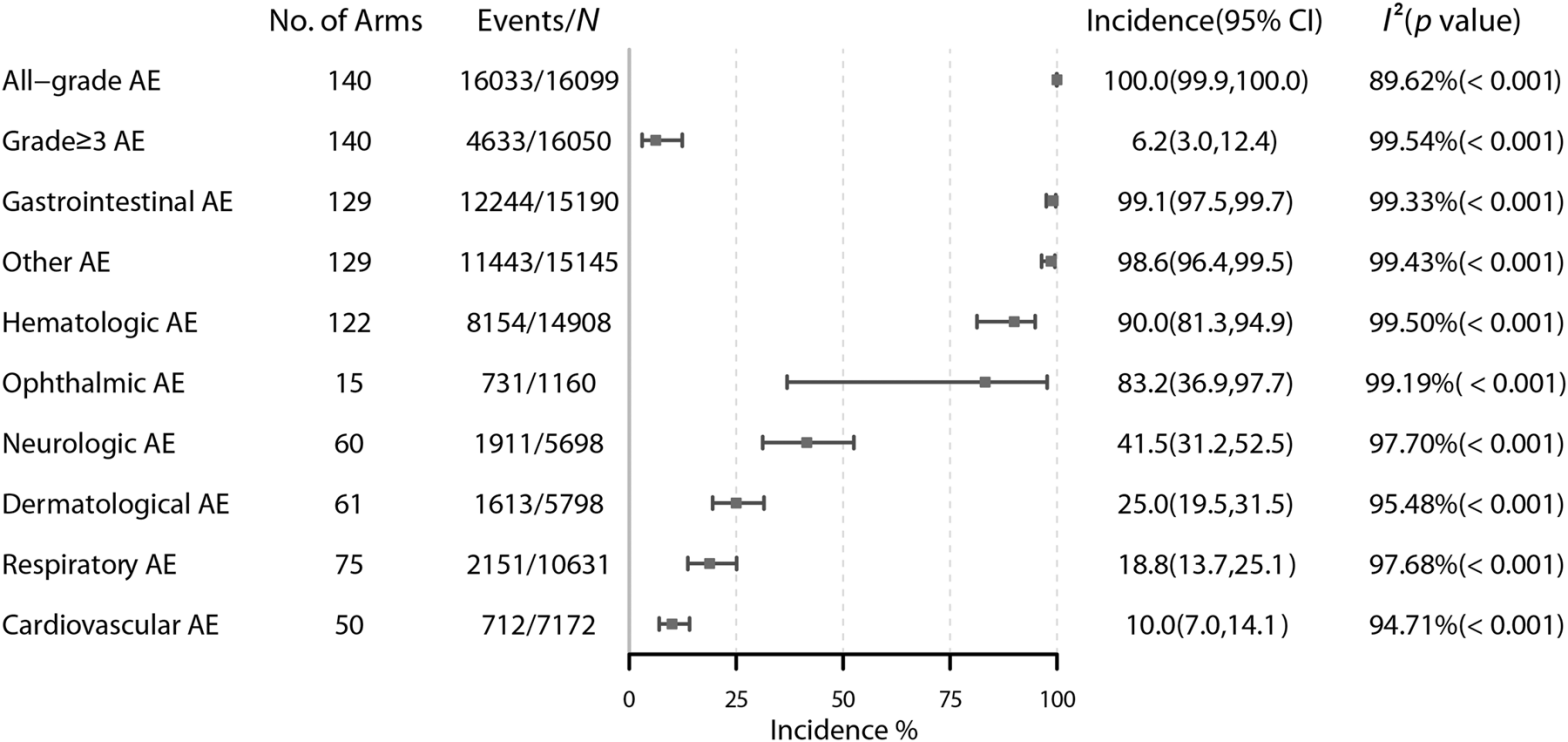
Overall incidence of adverse events.

Figure 2 displays the overall incidence of any‐grade and grade ≥ 3 AEs across various studies. The overall incidence of any‐grade treatment‐related AEs was 100.0% (95% CI: 99.9%–100.0%; *I* ^2^ = 89%) and that of grade ≥ 3 treatment‐related AEs was 6.2% (95% CI: 3.0%–12.4%; *I*² = 99%). A random‐effects model was used for analyses when *p* ≤ 0.10 or when the *I* ^2^ statistic indicated >50% study heterogeneity. According to body systems, the most frequently reported any‐grade AEs were gastrointestinal AEs (99.1%, 95% CI: 97.5%–99.7%), followed by other (98.6%, 95% CI: 96.4%–99.5%), hematologic (90.0%, 95% CI: 81.3%–94.9%), ophthalmic (83.2%, 95% CI: 36.9%–97.7%), neurologic (41.5%, 95% CI: 31.2%–52.5%), dermatological (25.0%, 95% CI: 19.5%–31.5%), respiratory (18.8%, 95% CI: 13.7%–25.1%), and cardiovascular (10.0%, 95% CI: 7.0%–14.1%) AEs.

**Figure 3:**
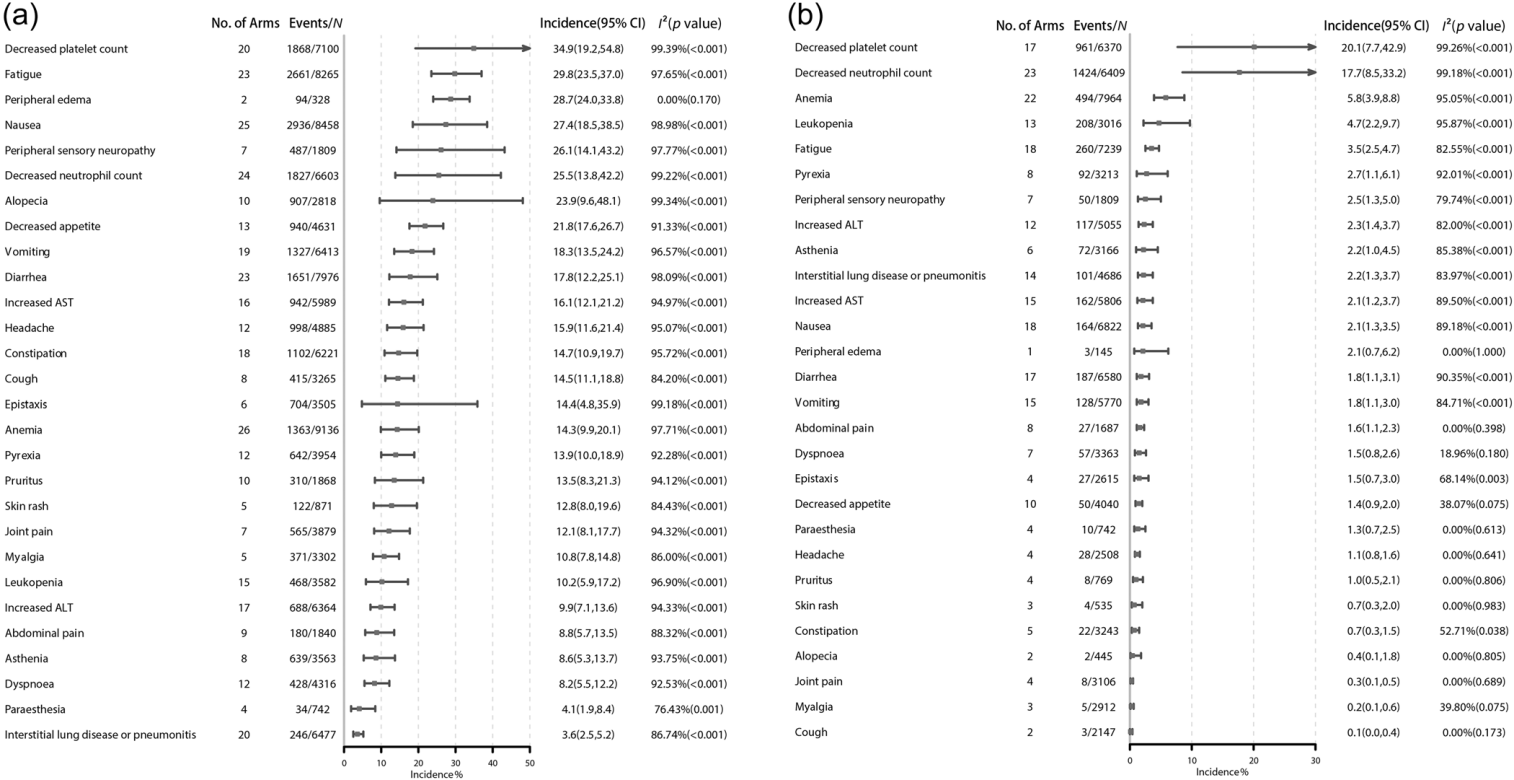
Incidence of most frequently reported adverse events. (a) The most common all grade adverse events and (b) the most common grade ≥ 3 adverse events.

The most commonly reported all‐grade AEs occurring in ≥20% of patients were decreased platelet count (34.9%, 95% CI: 19.2%–54.8%), fatigue (29.8%, 95% CI: 23.5%–37.0%), peripheral edema (28.7%, 95% CI: 24.0%–33.8%), nausea (27.4%, 95% CI: 18.5%–38.5%), peripheral sensory neuropathy (26.1%, 95% CI: 14.1%–43.2%), decreased neutrophil count (25.5%, 95% CI: 13.8%–42.2%), alopecia (23.9%, 95% CI: 9.6%–48.1%), and decreased appetite (21.8%, 95% CI: 17.6%–26.7%) (Figure 3). AEs occurring in 15%–20% of patients were vomiting (18.3%, 95% CI: 13.5%–24.2%), diarrhea (17.8%, 95% CI: 12.2%–25.1%), increased aspartate transaminase levels (16.1%, 95% CI: 12.1%–21.2%), and headache (15.9%, 95% CI: 11.6%–21.4%). The most frequently reported grade ≥ 3 AE was decreased platelet count (20.1%, 95% CI: 7.7%–42.9%), followed by decreased neutrophil count (17.7%, 95% CI: 8.5%–33.2%), anemia (5.8%, 95% CI: 3.9%–8.8%), leukopenia (4.7%, 95% CI: 2.2%–9.7%), and fatigue (3.5%, 95% CI: 2.5%–4.7%).

**Figure 4:**
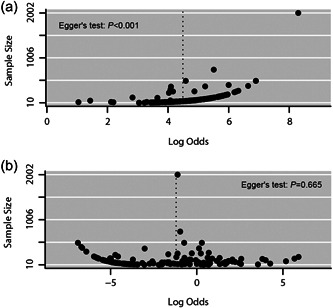
Funnel plots. (a) All‐grade adverse events and (b) grade ≥ 3 adverse events.

There was obvious asymmetry in the modified funnel plots for all‐grade AEs (Figure 4a) with *p* < 0.001 in Egger's test; the incidence of all‐grade AEs was 97.9% (95% CI: 97.3%–98.3%) after trim and fill correction. No obvious asymmetry was observed in the modified funnel plots for grade ≥ 3 AEs (Figure 4b) (*p* = 0.665 in Egger's test). Because obvious heterogeneity was observed in the incidence of all‐grade and Gade ≥ 3 AEs across arms, we performed subgroup analyses to explore the source of heterogeneity.

### 3.2 Incidence of special interest AEs related to ADCs

We analyzed AEs of special interest related to ADCs, including ophthalmic toxicity, hepatotoxicity, peripheral neuropathy, severely decreased neutrophil count, left cardiac dysfunction, diarrhea, dermatological toxicity, interstitial lung disease, and pneumonitis. In total, 106 (76.26%) trials reporting the incidence of ADC‐related AEs were included.

**Figure 5:**
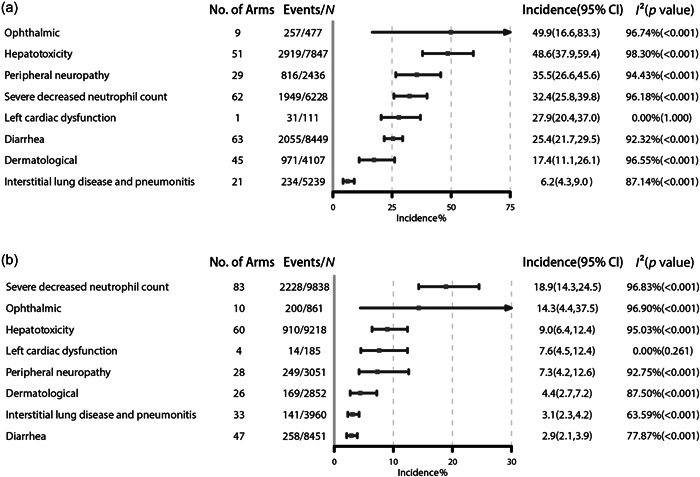
Incidence of antibody‐drug conjugate‐related special interest adverse events. (a) Most frequently reported all‐grade adverse events and (b) most frequently reported grade ≥ 3 adverse events.

The most frequently reported any‐grade AE was ophthalmic toxicity (49.9%, 95% CI: 16.6%–83.3%), followed by hepatotoxicity (48.6%, 95% CI: 37.9%–59.4%), peripheral neuropathy (35.5%, 95% CI: 26.6%–45.6%), severely decreased neutrophil count (32.4%, 95% CI: 25.8%–39.8%), left cardiac dysfunction (27.9%, 95% CI: 20.4%–37.0%), diarrhea (25.4%, 95% CI: 21.7%–29.5%), dermatological reactions (17.4%, 95% CI: 11.1%–26.1%), and interstitial lung disease and pneumonitis (6.2%, 95% CI: 4.3%–9.0%) (Figure 5a). The most frequent grade ≥ 3 AEs were severely decreased neutrophil count (18.9%, 95% CI: 14.3%–24.5%), ophthalmic toxicity (14.3%, 95% CI: 4.4%–37.5%), hepatotoxicity (9.0%, 95% CI: 6.4%–12.4%), left cardiac dysfunction (7.6%, 95% CI: 4.5%–12.4%), peripheral neuropathy (7.3%, 95% CI: 4.2%–12.6%), dermatological reactions (4.4%, 95% CI: 2.7%–7.2%), interstitial lung disease and pneumonitis (3.1%, 95% CI: 2.3%–4.2%), and diarrhea (2.9%, 95% CI: 2.1%–3.9%) (Figure 5b).

### 3.3 Incidence of treatment discontinuation and treatment‐related deaths

The incidence of treatment‐related AEs leading to ADC treatment discontinuation was 11.2% (95% CI: 9.3%–13.3%; 1,547/12,974 participants) (Supporting Information: eFigure 1). Forty‐three studies reported the incidence of treatment‐related deaths. Overall, 129 treatment‐related deaths were observed, with an overall incidence of 0.4% (95% CI: 0.2%–0.7%; 129/11,558 participants). The most common cause of treatment‐related death (*n* = 129) was interstitial lung disease (28, 21.70%), followed by decreased platelet count (15, 11.63%), dyspnea (9, 6.98%), decreased neutrophil count (8, 6.20%), and pneumonia (5, 3.88%) (Table 2). Respiratory causes (45, 34.88%) accounted for almost 50% of all treatment‐related deaths. Other common causes included gastrointestinal (7, 5.43%), cardiovascular (5, 3.88%), hematologic (28, 21.71%), infectious (8, 6.20%), and urinary (2, 1.55%) causes.

**Table 2:** Causes of 129 treatment‐related deaths in clinical trials of antibody‐drug conjugates.

Cause of death	No. (%)
Total death	129 (100)
Respiratory (*n* = 45)	
Interstitial lung disease	28 (21.70)
Dyspnea	9 (6.98)
Pneumonia	5 (3.88)
Respiratory failure	3 (2.33)
Gastrointestinal (*n* = 7)	
Acute hepatic failure	4 (3.10)
Hepatotoxicity	3 (2.33)
Cardiovascular (*n* = 5)	
Myocardial infarction	3 (2.33)
Cardiac arrest	2 (1.55)
Hematologic (*n* = 28)	
Decreased platelet count	15 (11.63)
Decreased neutrophil count	8 (6.20)
Leukopenia	5 (3.88)
Infectious (*n* = 8)	
Sepsis	5 (3.88)
Septic shock	3 (2.33)
Urinary (*n* = 2)	
Hemolytic uremic syndrome	2 (1.55)
Cerebrovascular (*n* = 1)	
Cerebral hemorrhage	1 (0.78)
Other (*n* = 22)	
Hypokalemia	3 (2.33)
Urinary tract obstruction	2 (1.55)
Physical pain	3 (2.33)
Multiorgan failure	5 (3.88)
Infusion‐related reactions	2 (1.55)
Musculoskeletal and connective‐tissue	1 (0.78)
Dehydration	1 (0.78)
Toxic epidermal necrolysis	2 (1.55)
General physical health deterioration	2 (1.55)
Severe skin reaction	1 (0.78)
Unspecified (*n* = 11)	
Neoplasms	2 (1.55)
Respiratory disorders	1 (0.78)
Malignant neoplasm progression	2 (1.55)
Unknown	6 (4.70)

### 3.4 Subgroup analysis of overall AE incidence according to cancer type

The overall incidence of AEs was evaluated according to the type of cancer, including solid tumors (breast, gastric, and cervical cancers, head and neck squamous cell carcinoma, urothelial carcinoma, and lung cancer) and hematological malignancies (lymphoma, leukemia, and multiple myeloma). Among solid tumors, the most frequently reported any‐grade AEs were gastrointestinal AEs (100.0%, 95% CI: 99.8%–100.0%), followed by other (99.7%, 95% CI: 97.9%–99.9%), hematologic (94.3%, 95% CI: 84.7%–98.0%), and neurologic (31.8%, 95% CI: 21.5%–44.3%) AEs, and grade ≥ 3 AEs accounted for 3.5% of all AEs (95% CI: 1.2%–9.3%). Among hematological malignancies, the most frequently reported any‐grade AE was ophthalmic toxicity (99.5%, 95% CI: 46.8%–100.0%), followed by other (95.8%, 95% CI: 88.1%–98.6%), gastrointestinal (95.5%, 95% CI: 88.9%–98.3%), hematologic (83.3%, 95% CI: 65.9%–92.8%), and neurologic (51.1%, 95% CI: 34.7%–67.2%) AEs; grade ≥ 3 AEs accounted for 10.6% of all AEs (95% CI: 3.9%–25.7%) (Supporting Information: eFigure 2A,B).

### 3.5 Subgroup analysis of overall AE incidence based on ADC type

All ADC drugs were associated with >98.0% of any‐grade AEs, but these ADCs showed remarkable differences in the incidence of grade ≥ 3 AEs: 98.4% (95% CI: 87.6%–99.8%) for loncastuximab tesirine, 44.0% (95% CI: 10.1%–84.6%) for gemtuzumab ozogamicin, 36.4% (95% CI: 17.4%–60.9%) for disitamab vedotin, 33.8% (95% CI: 9.3%–71.8%) for inotuzumab ozogamicin, and 10.5% (95% CI: 0.9%–59.0%) for sacituzumab govitecan, whereas the incidence of grade ≥ 3 AEs for other ADCs was <10% (Supporting Information: eFigure 3A,B).

### 3.6 Subgroup analysis of overall AE incidence based on ADC components

ADCs are composed of three components: antibody, linker, and payload. The overall incidence of AEs was evaluated according to three types (HER‐2, TROP‐2, and nectin‐4) of antibodies in solid tumors, and the incidence of grade ≥ 3 AEs was evaluated according to nine types of antibodies, two types of linkers (cleavable and noncleavable), and five types of payload (camptothecin, DM1, MMAE, MMAF, and SN‐38) Supporting Information: eTables 3 and 4). All antibodies were associated with >98.0% of any‐grade AEs but showed remarkable differences in the incidence of grade ≥ 3 AEs (98.4%, 95% CI: 87.6%–99.8%) for CD19 and 44.0% (95% CI: 10.1%–84.6%) for CD33. The incidence of any‐grade AEs for cleavable ADC drugs was 100.0% (95% CI: 99.8%–100.0%) and the incidence of grade ≥ 3 AEs was 7.9% (95% CI: 3.5%–16.6%), whereas the respective incidences for noncleavable ADC drugs were 99.8% (95% CI: 99.4%–100.0%) and 2.2% (95% CI: 0.4%–10.7%). We also evaluated the incidences of any‐grade and grade ≥ 3 AEs for payloads. All payloads were associated with >99.0% of any‐grade AEs, and the incidences of grade ≥ 3 AEs were 98.4% (95% CI: 87.6%–99.8%) for PBD dimer SG3199 antibody and 39.1% (95% CI: 15.3%–69.6%) for calicheamicin antibody. The main payloads causing respiratory, dermatological, neurotoxic, and ophthalmic AEs were SN‐38 (80.9%, 95% CI: 2.2%–99.9%), PBD dimer SG3199 (53.9%, 95% CI: 36.0%–70.8%), MMAE (52.1%, 95% CI: 41.0%–62.9%), and MMAF (99.9%, 95% CI: 30.8%–100.0%). Most payloads caused gastrointestinal and other AEs, but had low cardiovascular toxicity (Supporting Information: eFigures 4, 5, and 6).

## 4 DISCUSSION

Several ADCs have been approved by drug administrations and have been widely used in clinical trials and emerging studies worldwide. Despite the increased focus by clinicians and researchers on the safety of ADCs, however, a comprehensive AE profile for ADCs remains to be clearly defined. To the best of our knowledge, the current study was the first to analyze the incidence of ADC‐related AEs in studies published to date. The results showed that patients treated with ADCs had a high overall incidence of any‐grade AEs but a relatively low incidence of grade ≥ 3 AEs. Several ADC‐related special interest AEs were noted, as well as differences in the incidences of any‐grade and grade ≥ 3 AEs among different cancer types, ADC types, and ADC components.

ADCs are typically composed of an antibody and a cytotoxic payload linked via a chemical linker [8]. The specific cytotoxic payload can selectively target cancer cells through delivery of a high‐affinity antibody. In principle, ADC‐related AEs can be induced by any component of the drug [157, 158]. The main mechanisms responsible for adverse effects include suboptimal monoclonal antibody specificity, target antigen expression on normal cells, early cleavage of the linker, drug immunogenicity, and binding to Fc and mannose receptors. Off‐target effects of cytotoxic payloads associated with unwanted bystander effects are thought to be the primary cause of ADC‐related AEs [1, 159, 160].

To the best of our knowledge, only one previous meta‐analysis, including 70 publications, has evaluated the incidence of ADC‐related AEs, which concluded that most grade 3 and 4 ADC‐associated toxicities were related to the payload [161]. However, that study had several limitations. First, it primarily focused on the incidence of grade ≥ 3 AEs related to payload class, rather than summarizing and comprehensively evaluating any‐grade AEs. Second, it included agents that were not yet in the market. Third, it did not analyze other ADC components or different populations in depth, and was published 5 years ago. Fourth, it only analyzed some AEs and missed several important ones, such as pneumonitis, hepatotoxicity, and ophthalmic toxicity, which are important with respect to ADCs. There is thus a need for a comprehensive analysis of all common AEs caused by drug administration‐approved ADCs, including those previously reported in RCTs [162].

In the present study, the overall incidence of any‐grade AEs was 100.0% (95% CI: 99.9%–100.0%) and that of grade ≥ 3 AEs was 6.2% (95% CI: 3.0%–12.4%). Decreased platelet count was the most common any‐grade AE (34.9%) and the most common grade ≥ 3 AE (20.1%). Although less likely to be severe at presentation, the incidence of decreased platelet count was relatively high and is thus worth disclosing to patients. Fatigue, peripheral edema, nausea, peripheral sensory neuropathy, decreased neutrophil count, alopecia, and decreased appetite were the next most common any‐grade AEs; however, the likelihood of patients experiencing serious manifestations of these AEs is relatively low. Decreased neutrophil count, anemia, leukopenia, and fatigue are common grade ≥ 3 AEs, and patients should not be overly concerned about these AEs related to the cytotoxicity induced by ADC drug linkage.

Our meta‐analysis also identified some special interest ADC‐related AEs that were less associated with chemotherapy or targeted agents but more common among ADC drugs, including hepatotoxicity, ophthalmic toxicity, dermatological reactions, left cardiac dysfunction, interstitial lung disease and pneumonitis, diarrhea, severely decreased neutrophil count, and peripheral neuropathy. Ophthalmic toxicities caused by ADC drugs are mainly related to the payload (MMAF), which can damage corneal epithelial cells [104, 163]. Furthermore, ADCs were found to cause hepatotoxicity associated with a calicheamicin payload, increasing the incidence of liver injury or veno‐occlusive disease, while MMAFs led to the development of peripheral neuropathy and neutropenic AEs. Moreover, gemtuzumab ozogamicin causes accumulation of antitoxin conjugates in liver cells, resulting in calicheamicin‐induced damage [164, 165]. Trastuzumab deruxtecan comprises an anti‐HER2 antibody, tetrapeptide‐based cutout linkers, and topoisomerase I inhibitor loads, and interstitial pneumonia caused by this drug is an AE of particular concern compared with other ADC drugs. HER2 expression in the bronchial and fine bronchial epithelium of the lung may be associated with the development of trastuzumab deruxtecan‐associated interstitial lung disease [11, 166]. In a recent study on crab‐eating monkeys, immunohistochemical analysis confirmed that trastuzumab deruxtecan was localized mainly in alveolar macrophages but not in lung epithelial cells [167]. Nontarget trastuzumab deruxtecan uptake by alveolar macrophages was also observed in animal models, suggesting that lung tissue payload release is involved in the development of interstitial lung disease caused by this drug [167, 168]. However, further studies are needed to determine the risk factors and mechanisms involved in the development of trastuzumab deruxtecan‐associated interstitial lung disease.

This meta‐analysis indicated several fatal toxic events of ADCs, with an overall mortality of 0.4%. Interstitial lung disease was the most common cause of ADC‐related death, followed by decreased platelet count, dyspnea, and decreased neutrophil count. Recognizing the profile of fatal toxic events is crucial for facilitating their early detection and effectively managing these events. Notably, although clinicians have been familiar with relatively common fatal toxic events such as pneumonitis, some rare fatal events, including diarrhea and dermatological reactions, should also be routinely screened.

Subgroup analyses of the overall AE incidence based on cancer type, ADC type, and ADC components indicated significant differences in all cases. Overall, nine types of cancers were treated with 14 types of ADCs. Among solid tumors, the most frequently reported any‐grade AEs were gastrointestinal and hematologic AEs. Among patients with hematological malignancies, the most frequently reported any‐grade AEs were ophthalmic and gastrointestinal AEs. grade ≥ 3 AEs accounted for <11% of all AEs for both solid and hematological tumors. The choice of ADC drug target and antibody quality determine the affinity of the ADC for tumor cells, the type of linker determines the stability of the drug, and the choice of payload determines the AEs of ADCs [169]. Eleven target types and their monoclonal antibodies were used in these ADCs. The toxicity of ADCs may be related to any of their components. For target analysis, the antibody binds precisely to the antigen in a delivery‐specific manner to kill tumor cells, while avoiding binding to normal cells to reduce the resulting toxicity [8]. Causes of antibody‐induced toxicity include low antibody affinity, insufficient antigen expression on tumor cells, and lack of internalization upon binding. For example, HER‐2 protein is predominantly located on the myocardial transverse myocardium, and HER‐2 and its downstream signaling pathways are closely associated with cardiac function, further inducing cardiotoxicity. Among the ADCs used, trastuzumab showed a high risk of cardiotoxicity [170, 171]. Labetuzumab govitecan is an ADC against carcinoembryonic antigen‐associated cell adhesion molecule 5 and SN‐38, the active metabolites of the antineoplastic drug irinotecan. ADC drugs bind to tumor surface targets through antibodies, and then enter cells through internalization to kill tumor cells. Because Labetuzumab govitecan lacks internalization, this ADC drug will produce certain toxicity after entering Phase I studies [8, 172]. Most studies have shown that the toxic effects caused by ADCs in clinical settings are mainly related to the payload.

Different ADC components have markedly distinct AE spectra, as observed in the comparison between noncleavable and cleavable linkers. Noncleavable linkers are stable in plasma but have limited efficacy at the target cell, while cleavable linkers are unstable in plasma, leading to off‐target toxicity, but have higher efficacy at the target cell [8, 173]. Our meta‐analysis showed that ADCs with cleavable linkers resulted in a higher overall incidence of any‐grade and grade ≥ 3 AEs compared with noncleavable linkers. ADCs with both cleavable and noncleavable linkers are associated with a high incidence of gastrointestinal and hematological AEs; however, ADCs with only cleavable linkers mainly cause neurological AEs while ADCs with noncleavable linkers are mainly associated with ophthalmic AEs. We did not carry out any further investigation to determine if specific AEs were more common in particular cancer types (e.g., pneumonitis in lung cancer or colitis in gastrointestinal cancer).

Payload analysis revealed the highest difference in the overall incidence of any‐grade and grade ≥ 3 AEs. Most payloads used in ADCs are highly cytotoxic and mediate the AEs of most ADCs. Our results showed that various payload was associated with an increased risk of any‐grade AEs, and PBD dimer SG3199 and calicheamicin were associated with increased risks of grade ≥ 3 AEs. SN‐38, PBD dimer SG3199, and MMAEs cause adverse respiratory effects, such as pneumonia, skin toxicity, and neurotoxicity, while deruxtecan, camptothecin, SN‐38, and calicheamicin mainly cause hematologic toxicity and MMAF causes ophthalmic toxicity. Most payloads can cause digestive and other toxicities, but have low cardiovascular toxicity.

This study had some limitations. First, the diagnosis of AEs was evaluated by the original investigators in the different trials with no standardized or uniform definitions and/or classification. Moreover, the judgment of whether or not an AE was associated with an ADC might be susceptible to bias. There may also be overlap in the extraction process of adverse reaction data, such as elevated liver function indicators, hepatotoxicity, and ADC‐related hepatitis. Second, there was heterogeneity among the included trials with regard to targeted drugs, linkers, payload of ADCs, enrolled population, and number of treatment lines. We conducted multiple subgroup analyses to validate the results; however the limited number of included studies meant that we could not analyze several subgroups, for example, whether AEs were caused by a particular ADC component or by a combination of drug and disease factors. Finally, the number of trials of several ADCs was limited. Additional studies with larger sample sizes are therefore needed to confirm our findings.

## 5 CONCLUSIONS

This systematic review and meta‐analysis summarized the profile of AEs associated with ADC use and the common causes of treatment‐related death. ADCs were associated with a high incidence of any‐grade AEs but a relatively low incidence of grade ≥ 3 AEs. The type of cancer, type of ADC, and ADC components were associated with varying profiles and incidences of AEs. These findings regarding ADC‐related AEs may be useful for clinicians and researchers.

## AUTHOR CONTRIBUTIONS

**Jinming Li**, **Guoshuang Shen**, and **Zhen Liu**: Data curation (equal); writing—original draft (equal). **Yaobang Liu**: Formal analysis (equal); writing—review and editing (equal). **Miaozhou Wang**, **Fuxing Zhao**, and **Dengfeng Ren**: Conceptualization (equal); methodology (equal). **Qiqi Xie** and **Zitao Li**: Data curation (supporting); software (supporting). **Zhilin Liu**: Resources (supporting); validation (supporting). **Yi Zhao**: Investigation (lead); supervision (lead). **Fei Ma**: Project administration (lead); writing—review and editing (lead). **Xinlan Liu** and **Jiuda Zhao**: Conceptualization (lead); project administration (lead).

## CONFLICT OF INTEREST STATEMENT

Professor Fei Ma and Jiuda Zhao are the members of the *Cancer Innovation* Editorial Board. To minimize bias, they were excluded from all editorial decision‐making related to the acceptance of this article for publication. The remaining authors declare no conflict of interest.

## ETHICS STATEMENT

This study used statistical methods to quantitatively synthesize the results of several independent clinical studies addressing the same clinical problem. The study did not involve human participation and was therefore exempt from the need for IRB review.

## INFORMED CONSENT

Not applicable.

## Supporting information

eTable 1. Characteristics of components of antibody‐drug conjugates.

eTable 2. Search process.

eTable 3. Components of ADCs.

eTable 4. Incidence and types of treatment‐related adverse events according to cancer type and ADC component.

eFigure 1. Incidence of treatment discontinuation and treatment‐related deaths.

eFigure 2. Overall incidence of adverse event according to cancer type. (A) Incidence of adverse events of all grades in patients with solid tumors and hematologic malignancies; (B) incidence of adverse events of grade ≥3 in patients with solid tumors and hematologic malignancies.

eFigure 3. Overall incidence of adverse events based on ADC type. (A) Incidence of all grade adverse events based on ADC type; (B) incidence of grade ≥3 adverse events based on ADC type.

eFigure 4. Overall incidence of adverse events based on antibody. (A) Incidence of all grade adverse events based on antibody; (B) incidence of grade ≥3 adverse events based on antibody.

eFigure 5. Overall incidence of adverse events based on linker. (A) Incidence of all grades of adverse events based on linker; (B) incidence of grade ≥3 adverse events based on linker.

eFigure 6. Overall incidence of adverse events based on payload. (A) Incidence of all‐grade adverse events based on payload; (B) incidence of grade ≥3 adverse events based on payload.

Supporting information.

## Data Availability

The data used in the work is all open source data on the web.
