# Supplementary material for: Treatment‐related adverse events of antibody‐drug conjugates in clinical trials: A systematic review and meta‐analysis
Source: Cancer Innov. 2023 Oct 15;2(5):346–75. doi: 10.1002/cai2.97 (PMC10686142; doi:10.1002/cai2.97)
Supplement: Supplementary file 1 — eTable 1. Characteristics of components of antibody‐drug conjugates. [file CAI2-2-346-s010.docx]

eTable 1. The characteristics of component of ADC drugs.

| **ADCs drugs** | **Trade Name** | **Target** | **Year of initial FDA approval** | **Condition** |
| --- | --- | --- | --- | --- |
| Brentuximab vedotin | Adcetris | CD30 | 2011 | Relapsed  hairy leukemia and relapsed anaplastic large cell lymphoma |
| Ado-trastuzumab emtansine/T-DM1 | Kadcyla | HER2 | 2013 | HER2-positive metastatic breast cancer following treatment with trastuzumab and a maytansinoid |
| Inotuzumab ozogamicin | Besponsa | CD22 | 2017 | Relapsed or refractory CD22-positive B-cell precursor acute lymphoblastic leukemia |
| Gemtuzumab ozogamicin | Mylotarg | CD33 | 2017 | Relapsed acute myelogenous leukemia |
| Polatuzumab vedotin | Polivy | CD79b | 2019 | Relapsed or refractory diffuse large B-cell lymphoma |
| Fam-trastuzumab deruxtecan-nxki/ (T-DXd)/(DS-8201a) | Enhertu | HER-2 | 2019 | Adult patients with unresectable or metastatic HER2-positive breast cancer who have received two or more prior anti-HER2 based regimens |
| Sacituzumab govitecan/IMMUNO-132/HRS7-SN38 | TrodelvyTM | TROP-2 | 2020 | Adult patients with metastatic triple-negative breast cancer who have received at least two prior therapies for patients with relapsed or refractory metastatic disease |
| Enfortumab vedotin | Padcevtm | nectin-4 | 2019 | Adult patients with locally advanced or metastatic urothelial cancer who have received a PD-1 or PD-L1 inhibitor, and a Pt-containing therapy |
| Tisotumab vedotin-tftv/TF-011-MMAE/HUMAX-TF-ADC | Tivdak | TF(CD142) | 2021 | Recurrent or metastatic cervical cancer |
| Disitamab Vedotin（RC48） | Aidixi | HER-2 | 2018 | Locally advanced or metastatic HER2 overexpressing gastric cancer (including adenocarcinoma of the gastroesophageal junction |
| Belantamab Mafodotin-blmf | Blenrep™ | BCMA | 2020 | Adult patients with relapsed or refractory multiple myeloma |
| [Moxetumomab pasudotox](https://en.wikipedia.org/wiki/Moxetumomab_pasudotox) | Lumoxiti | CD22 | 2018 | Adults with relapsed or refractory hairy cell leukemia |
| [Loncastuximab tesirine-lpyl](https://en.wikipedia.org/wiki/Loncastuximab_tesirine) | Zynlonta | CD19 | 2021 | Large B-cell lymphoma |
| RM-1929 | Akalux | EGFR | 2020 | Recurrent head and neck squamous cell carcinoma of the head and neck that has failed 2nd line therapy |

Abbreviations: ADC, Antibody-Drug Conjugates; HER2, human epidermal growth factor receptor 2; PD-1, programmed death 1; PD-L1, programmed cell death-Ligand 1
