# Supplementary material for: Treatment‐related adverse events of antibody‐drug conjugates in clinical trials: A systematic review and meta‐analysis
Source: Cancer Innov. 2023 Oct 15;2(5):346–75. doi: 10.1002/cai2.97 (PMC10686142; doi:10.1002/cai2.97)
Supplement: Supplementary file 2 — eTable 2. Search process. [file CAI2-2-346-s005.docx]

eTable 2. Search process.

| **Database** | **Keywords** |
| --- | --- |
| **PubMed** |  |
| #1 | "Disitamab Vedotin"[Title/Abstract] OR "RC48"[Title/Abstract] OR "Tisotumab vedotin"[Title/Abstract] OR "Enfortumab vedotin"[Title/Abstract] OR "Polatuzumab vedotin"[Title/Abstract] OR "Trastuzumab Deruxtecan"[Title/Abstract] OR "(DS-8201)"[Title/Abstract] OR "Sacituzumab Govitecan"[Title/Abstract] OR "IMMU-132"[Title/Abstract] OR "Moxetumomab pasudotox"[Title/Abstract] OR "CAT-8015"[Title/Abstract] OR "HA22"[Title/Abstract] OR "Inotuzumab ozogamicin"[Title/Abstract] OR "CMC-544"[Title/Abstract] OR "Brentuximab vedotin"[Title/Abstract] OR "Trastuzumab Emtansine"[Title/Abstract] OR "T-DM1"[Title/Abstract] OR "Belantamab mafodotin"[Title/Abstract] OR "GSK2857916"[Title/Abstract] OR "Loncastuximab tesirine"[Title/Abstract] OR "ADCT-402"[Title/Abstract] OR "RM‑1929"[Title/Abstract] OR "anti-EGFR–IR700 dye conjugate"[Title/Abstract] OR "Polatuzumab vedotin"[Title/Abstract] OR "antibody-drug conjugates"[Title/Abstract] OR "ADC"[Title/Abstract] |
| #2 | "combination"[Title/Abstract] OR "combine"[Title/Abstract] OR "combined"[Title/Abstract] OR "plus"[Title/Abstract] OR "addition"[Title/Abstract] OR "concurrent"[Title/Abstract] OR "concomitant"[Title/Abstract] |
| #3 | "adverse events"[Title/Abstract] OR "toxicity"[Title/Abstract] OR "side effects"[Title/Abstract] OR " adverse  reaction"[Title/Abstract] |
| #4 | "cancer"[Title/Abstract] OR "neoplasm"[Title/Abstract] OR "tumor"[Title/Abstract] OR "neoplasia"[Title/Abstract] OR "malignancy"[Title/Abstract] |
| #5 | "clinical trials as topic"[MeSH Terms] OR "clinical trial"[Publication Type] OR "phase"[Title/Abstract] OR "clinical trials"[Title/Abstract] |
| #6 | ("2001/01/01"[Date - Publication] : "2022/10/31"[Date - Publication]) |
| #7 | #1 AND #2 AND #3 AND #4 AND #5 AND #6 |
| #8 | "review"[Article type] OR "meta"[Title] OR "meta-analysis"[Title] OR "protocol"[Title] |
| #9 | #7 NOT #8 |
| **EMBASE** |  |
| #1 | (Disitamab Vedotin OR RC48 OR Tisotumab vedotin OR Enfortumab vedotin OR Polatuzumab vedotin OR Trastuzumab Deruxtecan OR DS-8201 OR Sacituzumab Govitecan OR IMMU-132 OR Moxetumomab pasudotox OR CAT-8015 OR HA22 OR Inotuzumab ozogamicin OR CMC-544 OR Brentuximab vedotin OR Trastuzumab Emtansine OR T-DM1 OR Belantamab mafodotin OR GSK2857916 OR Loncastuximab tesirine OR ADCT-402 OR RM‑1929 OR anti-EGFR–IR700 dye conjugate OR Polatuzumab vedotin OR antibody-drug conjugates OR ADC):ti,ab,kw |
| #2 | (combination OR combine OR combined OR plus OR addition OR concurrent OR concomitant):ti,ab,kw |
| #3 | trial/exp OR 'clinical trials'/exp OR 'phase 1 clinical trial'/exp OR 'phase 2 clinical trial'/exp OR 'phase 3 clinical trial'/exp |
| #4 | [article]/lim OR [article in press]/lim |
| #5 | [humans]/lim |
| #6 | #1 AND #2 AND #3 AND #4 AND #5 |
| **CENTRAL** |  |
| #1 | (Disitamab Vedotin OR RC48 OR Tisotumab vedotin OR Enfortumab vedotin OR Polatuzumab vedotin OR Trastuzumab Deruxtecan OR DS-8201 OR Sacituzumab Govitecan OR IMMU-132 OR Moxetumomab pasudotox OR CAT-8015 OR HA22 OR Inotuzumab ozogamicin OR CMC-544 OR Brentuximab vedotin OR Trastuzumab Emtansine OR T-DM1 OR Belantamab mafodotin OR GSK2857916 OR Loncastuximab tesirine OR ADCT-402 OR RM‑1929 OR anti-EGFR–IR700 dye conjugate OR Polatuzumab vedotin OR antibody-drug conjugates OR ADC) |
| #2 | (combination OR combine OR combined OR plus OR addition OR concurrent OR concomitant):ti,ab,kw |
| #3 | (‘clinical trials as topic' OR trial):ti,ab,kw |
| #4 | ("conference" OR "review"):pt |
| #5 | #1 AND #2 AND #3 NOT #4 |
| #6 | Publication date: Between Jan 2001 and October 2022 |
