# Supplementary material for: Treatment‐related adverse events of antibody‐drug conjugates in clinical trials: A systematic review and meta‐analysis
Source: Cancer Innov. 2023 Oct 15;2(5):346–75. doi: 10.1002/cai2.97 (PMC10686142; doi:10.1002/cai2.97)
Supplement: Supplementary file 3 — eTable 3. Components of ADCs. [file CAI2-2-346-s004.docx]

eTable 3. Component of Antibody-Drug Conjugates.

| **Antibody-Drug Conjugates** | **Target** | **Linker** | **Payload** |
| --- | --- | --- | --- |
| Disitamab Vedotin (RC48) | HER-2 | Cleavable | MMAE |
| Tisotumab vedotin | TF(CD142) | Cleavable | MMAE |
| Enfortumab vedotin | Nectin-4 | Cleavable | MMAE |
| Polatuzumab vedotin | CD79b | Cleavable | MMAE |
| Trastuzumab Deruxtecan (DS-8201) | HER-2 | Cleavable | Deruxtecan |
| Sacituzumab Govitecan (IMMU-132) | TROP-2 | Cleavable | SN-38 |
| Moxetumomab pasudotox (CAT-8015, HA22) | CD22 | Cleavable | MMAE |
| Inotuzumab ozogamicin (CMC-544) | CD22 | Cleavable | Calicheamicin |
| Brentuximab vedotin | CD30 | Cleavable | MMAE |
| Trastuzumab Emtansine(T-DM1) | HER2 | Non-cleavable | DM1 |
| Belantamab mafodotin (GSK2857916) | BCMA | Non-cleavable | MMAF |
| Loncastuximab tesirine (ADCT-402) | CD19 | Cleavable | PBD dimer SG3199 |
| RM‑1929 (anti-EGFR–IR700 dye conjugate) | EGFR | Cleavable | IRDye700DX |
| Polatuzumab vedotin | CD79b | Cleavable | MMAE |
