# Supplementary material for: Treatment‐related adverse events of antibody‐drug conjugates in clinical trials: A systematic review and meta‐analysis
Source: Cancer Innov. 2023 Oct 15;2(5):346–75. doi: 10.1002/cai2.97 (PMC10686142; doi:10.1002/cai2.97)
Supplement: Supplementary file 4 — eTable 4. Incidence and types of treatment‐related adverse events according to cancer type and ADC component. [file CAI2-2-346-s011.docx]

eTable 4. Incidence and types of treatment-related according to cancer type and component.

| Variables, no. (%) | Cancer type | | | | | | | Component | | | | | | |
| --- | --- | --- | --- | --- | --- | --- | --- | --- | --- | --- | --- | --- | --- | --- |
|  | Gastrointestinal cancer (n =496) | Breast cancer  (n =6,655) | Urothelial carcinoma (n =1,852 ) | NSCLC or colorectal cancer or other solid cancer (n =195 ) | Head and Neck Squamous Cell Carcinoma (n = 61) | Uterine Cancer (n=1,108) | Hematological cancer (n=4,033 ) | Antibody | | Linker | | payload | | |
|  |  |  |  |  |  |  |  | For Soli（n=10,585） | For Hematological hematological malignancies（n=5,875） | Cleavable (n =10,488 ) | Non-cleavable (n =6,026) | DMEM(n=4,033) | Deruxtecan（Dxd)/ Camptothecin(n=1,886) | DM1  (n=5,380) |
| **Deaths** | **7（1.41）** | **23（0.35）** | **12（0.65）** | **5（2.56）** | **0** | **1（0.09）** | **81（2.01）** | **53(0.50)** | **89(1.51)** | **126(1.20)** | **16(0.27)** | **23(0.57)** | **65(3.45)** | **11(0.20)** |
| **Respiratory** | **28（5.65）** | **3861（58.01）** | **1（0.05）** | **30(15.38)** | **183(300)** |  | **216(5.36)** | **901（8.51）** | **561(9.55)** | **801(7.64)** | **661（10.97）** | **255(6.32)** | **633（33.56）** | **67（1.23）** |
| Interstitial lung  disease | 28（5.65） | 174（2.61） | 1（0.05） | 27（13.85） | 4（6.56） |  | 45（1.12） | 206(1.95) | 55(0.94) | 165(1.57) | 96(1.59) | 16(0.40) | 68(3.61) | 15(0.28) |
| Dyspnoea |  | 348（5.23） |  | 3（1.54） |  |  | 171（4.24） | 352(3.33) | 279(4.75) | 335(3.20) | 296(4.91) | 118(2.93) | 296(15.69) | 40(0.74) |
| Productive cough |  | 3339（50.17） |  |  | 179（293.44） |  |  | 343(3.24) | 227(3.86) | 301(2.87) | 269(4.46) | 121(3.00) | 269(14.26) | 12(0.22) |
| **Gastrointestinal** | **536（108.06）** | **6360（95.57）** | **1170（63.17）** | 200(102.56) | **6（9.84）** | **850(76.71)** | **1734(43.00)** | **8910（84.18）** | **2304(39.22)** | **7824(74.60)** | **3355（55.68）** | **2343（58.09）** | **3285（227.20）** | **371（6.90）** |
| Decreased appetite | 133（26.81） | 730（10.97） | 241（13.01） | 34（17.44） |  | 2（0.18） | 188（4.66） | 1239(11.71) | 270(4.60) | 1134(10.81) | 375(6.22) | 467(11.58) | 370(19.62) | 20(0.37) |
| Nausea | 137（27.62） | 2440（36.66） | 507（27.38） | 79（40.51） |  | 833（75.18） | 605（15） | 3276(30.95) | 765(13.02) | 2604(24.83) | 1402(23.27) | 769(19.07) | 1353(71.74) | 154(2.86) |
| Diarrhea | 71（14.31） | 1034（15.54） | 191（10.31） | 29（14.81） |  |  | 380（9.42） | 1699(16.05) | 476(8.10) | 1690(16.11) | 485(8.05) | 563(13.95) | 469(24.87 | 42(0.78) |
| Vomiting | 80（16.13） | 1035（15.55） | 231（12.47） | 36（18.46） |  |  | 257（6.37） | 1399(13.22) | 365(6.21) | 1295(12.35) | 469(7.78) | 212(5.27) | 469(24.87) | 93(1.73) |
| Constipation | 67（13.51） | 969（14.56） |  | 20（10.26） | 6（9.84） | 0 | 166（4.12） | 1096(10.35) | 267(4.54) | 772(7.36) | 591(9.81) | 177(4.39) | 591(31.34) | 43(0.80) |
| Abdominal pain | 48（9.68） | 133（2.00） |  |  |  | 15（1.35） | 113（2.80） | 180(1.70) | 136(2.31) | 288(2.75) | 28(0.46) | 138(3.42) | 28(1.48) | 11(0.20) |
| Anorexia |  | 19（0.29） |  | 2（1.03） |  |  | 25（0.62） | 21(0.20) | 25(0.43) | 41(0.39) | 5(0.08) | 17(0.42) | 5(0.27) | 8(0.15) |
| **Cardiovascular** |  | 102（1.53） |  |  | 0 |  | 95(2.36) | 110（1.04） | 78(1.33) | 78(0.74) | 110（1.83） | 81（2.00） | 110（5.83） | 5（0.09） |
| Uncontrolled  hypertension |  | 97（1.46） |  |  |  |  | 28（0.69） | 105(0.99) | 28(0.48) | 28(0.27) | 105(1.74) | 28(0.69) | 105(5.57) | 0 |
| Hypotension |  | 5（0.08） |  |  |  |  | 31（0.77） | 5(0.05) | 40(0.68) | 40(0.38) | 5(0.08) | 17(0.42) | 5(0.27) | 5(0.09) |
| Capillary leak syndrome |  |  |  |  |  |  | 26（0.64） | 0 | 0 | 0 | 0 | 26(0.64) | 0 | 0 |
| Tachycardia |  |  |  |  |  |  | 10（0.25） | 0 | 10(0.17) | 10(0.10) | 0 | 10(0.25) | 0 | 0 |
| **Neurologic** | **7（1.41）** | **280（4.21）** | **336（18.14）** |  | **9（14.75）** | **3（0.27）** | **526(13.04)** | **836（2.23）** | **397(6.76)** | **1089(10.38)** | **144（2.39）** | **1097（27.20）** | **144（7.64）** | **0** |
| Peripheral sensory neuropathy |  | 141（2.12） | 282（15.23） |  | 9（14.75） | 3（0.27） | 449（11.13） | 501(4.73) | 273(4.65) | 633(6.04) | 141(2.34) | 725(17.98) | 141(7.48) | 0 |
| Peripheral motor neuropathy | 7（1.41） | 52（0.78） |  |  |  |  | 67（1.66） | 0 | 74(1.26) | 74(0.71) | 0 | 74(1.83) | 0 | 0 |
| Hypoaesthesia |  | 1（0.02） | 54（2.92） |  |  |  | 1（0.02） | 249(2.35) | 41(0.70) | 289(2.18) | 1(0.02) | 289(7.17) | 1(0.05) | 0 |
| Neuropathy |  | 86（1.29） |  |  |  |  | 9（0.22） | 86(0.23) | 9(0.15) | 93(0.89) | 2(0.03) | 9(0.22) | 2(0.11) | 0 |
| **Hematologic** | **641（129.23）** | **3313（49.78）** | **535(28.89)** | 105(53.85) | **10(16.39)** | **226(20.40)** | **1836(45.52)** | **4860（45.91）** | **3997(68.03)** | **5958(56.81)** | **1584（26.29）** | **1578（39.13）** | **1288（68.29）** | **779（14.48）** |
| Leukopenia | 165（33.27） | 464（6.97） | 44（2.38） | 21（10.80） |  |  | 103（2.55） | 837(7.91) | 192(3.27) | 994(9.48) | 35(0.58) | 375(9.30) | 35(1.86) | 87(1.62) |
| Decreased neutrophil count | 187（37.70） | 864（12.98） | 169（9.13） | 32（16.41） |  | 3（0.27） | 593（14.71） | 1315(12.42) | 2174(37.00) | 2040(19.45) | 134(2.22) | 572(14.18) | 92(4.88) | 183(3.40) |
| Febrile neutropenia |  | 31（0.47） | 40（2.16） |  |  | 27（2.44） | 93（2.31） | 71(0.67) | 137(2.33) | 199(1.90) | 9(0.15) | 18(0.45) | 9(0.48) | 108(2.01) |
| Anemia | 169（34.07） | 889（13.36） | 261（14.09） | 32（16.41） | 10（16.39） | 196（17.69） | 374（9.27） | 1407(13.29) | 545(9.28) | 1512(14.42) | 440(7.30) | 273(6.77) | 335(17.76) | 118(2.19) |
| Decreased platelet count | 79（15.93） | 1007（15.13） | 9（0.49） | 20（10.26） |  |  | 542（13.44） | 1119(10.57) | 840(14.30) | 1038(9.90) | 921(15.28) | 293(7.27) | 785(41.62) | 220(4.19) |
| Decreased lymphocyte count | 41（8.27） | 58（0.87） | 12（0.65） |  |  |  | 131（3.25） | 111(1.05) | 109(1.86) | 175(1.67) | 45(0.75) | 47(1.17) | 32(1.70) | 63(1.17) |
| **Urinary** | **5（1.01）** | **205（3.08）** | **9(0.49)** |  |  | **11(0.99)** | **16(0.40)** | **233（2.20）** | **16(0.27)** | **85(0.81)** | **164（2.72）** | **38（0.94）** | **164（8.70）** | **0** |
| Urinary tract infection |  | 202（3.04） | 9（0.49） |  |  | 11（0.99） |  | 222(2.10) | 0 | 58(0.55) | 164（2.72） | 11(0.27) | 164(8.70) | 0 |
| Cystitis |  | 3（0.05） |  |  |  |  |  | 0 | 0 | 0 | 0 | 0 | 0 | 0 |
| Protein present in urine | 5（1.01） |  |  |  |  |  |  | 11(0.10) | 0 | 11(0.10) | 0 | 11(0.27) | 0 | 0 |
| Hemolytic uremic syndrome |  |  |  |  |  |  | 16（0.40） | 0 | 16(0.27) | 16(0.15) | 0 | 16(0.40) | 0 | 0 |
| **Ear, Nose and Throat System** | **0** | **653（9.81）** | **57(3.08)** | 6（3.08） | **4（6.56）** |  | **63(1.56)** | **882（8.33)** | **82(1.40)** | **317(3.02)** | **647（10.67）** | **114（2.83）** | **643（34.09）** | **4（0.07）** |
| Oropharyngeal pain |  | 2（0.03） |  |  | 4（6.56） |  | 20（0.50） | 6(0.06) | 20(0.34) | 24(0.23) | 2(0.03) | 20(0.50) | 2(0.11) | 0 |
| Rhinitis |  |  |  |  |  |  | 24（0.60） | 0 | 0 | 0 | 0 | 0 | 0 | 0 |
| Epistaxis |  | 635（9.54） |  | 6（3.08） |  |  | 4（0.10） | 677(6.40) | 4(0.07) | 40(0.38) | 641(10.64) | 36(0.89) | 641(33.98) | 4(0.07) |
| Nasopharyngitis |  |  |  |  |  |  | 15（0.37） | 0 | 58(0.99) | 58(0.55) | 0 | 58(1.44) | 0 | 0 |
| Dysgeusia |  | 16（0.24） | 57（3.08） |  |  |  |  | 199(1.88) | 0 | 195(1.86) | 4(0.07) | 0 | 0 | 0 |
| **Ophthalmic** | **0** | **3（0.05）** | **0** |  |  |  | **300(7.44)** | **17(0.16)** | **324(5.51)** | **20(0.19)** | **321（5.33）** | **20（0.50）** | **3（0.16）** | **0** |
| Dry eye |  | 3（0.05） |  |  |  |  | 20（0.50） | 16(0.15) | 20(0.34) | 13(0.12) | 23(0.38) | 13(0.32) | 3(0.16) | 0 |
| Corneal lesion |  |  |  |  |  |  | 190（4.71） | 0 | 214(3.64) | 0 | 214(3.55) | 0 | 0 | 0 |
| Vision blurred |  |  |  |  |  |  | 39（0.97） | 1(0.01) | 39(0.66) | 7(0.07) | 33(0.53) | 7(0.17) | 0 | 0 |
| Change in BCVA |  |  |  |  |  |  | 51（1.26） | 0 | 51(0.87) | 0 | 51(0.85) | 0 | 0 | 0 |
| **Infection** | **0** | **5（0.08）** |  |  |  |  | **21(0.52)** | **86(0.81)** | **137(2.33)** | **223(2.13)** | **0** | **3（0.07）** | **0** | **7（0.13）** |
| Cellulitis |  | 5（0.08） |  |  |  |  |  | 0 | 0 | 0 | 0 | 0 | 0 | 0 |
| Sepsis |  |  |  |  |  |  | 10（0.25） | 0 | 10(0.17) | 10(0.10) | 0 | 3(0.07) | 0 | 7(0.13) |
| Septic shock |  |  |  |  |  |  | 6（0.15） | 0 | 0 | 0 | 0 | 0 | 0 | 0 |
| infection |  |  |  |  |  |  | 5（0.12） | 86(0.81) | 127(2.16) | 213(2.03) | 0 |  |  |  |
| **Dermatological** | **41（8.27）** | **97（1.46）** | **235(12.69)** | 3（1.54） | **5（8.20）** | **0** | **387(9.60)** | **528(4.98)** | **543(9.24)** | **1058(10.09)** | **13（0.22）** | **639（15.84）** | **13（0.69）** | **19（0.35）** |
| Skin rash | 5（1.01） | 65（0.98） | 10（0.54） | 3（1.54） | 5（8.20） |  | 182（4.51） | 106(1.00) | 261(4.44) | 360(3.43) | 7(0.12) | 120(2.98) | 7(0.37) | 19(0.35) |
| Maculopapular rash |  |  | 83（4.48） |  |  |  | 59（1.46） | 116(1.10) | 91(1.55) | 207(1.97) | 0 | 126(3.12) | 0 | 0 |
| Dry skin |  | 15（0.23） | 34（1.84） |  |  |  |  | 77(0.73) | 0 | 77(0.73) | 0 | 60(1.49) | 0 | 0 |
| Pruritus | 36（7.26） | 17（0.26） | 108（5.83） |  |  |  | 146（3.62） | 229(2.16) | 191(3.25) | 414(3.95) | 6(0.10) | 333(8.26) | 6(0.32) | 0 |
| **Others** | **557（112.30）** | **5787（86.96）** | **1243(67.12)** | 123（63.08） | **20(32.79)** | **279(25.18)** | **2652(65.76)** | **8053(76.08)** | **3324(56.58)** | **7550(71.99)** | **3852（63.92）** | **3393（84.13）** | **3749（198.78）** | **514（9.55）** |
| Back pain | 16（3.23） | 59（0.89） |  |  |  |  | 86（2.13） | 40(0.38) | 52(0.89) | 98(0.93) | 19(0.32) | 84(2.08) | 19(1.01) | 0 |
| Joint pain | 15（3.02） | 539（8.10） |  |  |  |  | 126（3.12） | 560(5.29) | 126(2.14) | 175(1.67) | 511(8.48) | 138(3.42) | 511(27.09) | 9(9.55) |
| Physical pain |  | 36（0.54） |  |  |  |  | 28（0.69） | 57(0.54) | 28(0.48) | 60(0.57) | 25(0.41) | 53(1.31) | 25(1.33) | 0 |
| Fatigue | 131（26.41） | 2031（30.52） | 554（29.91） | 54（27.69） |  | 267（24.1） | 765（18.97） | 2913(27.52) | 971(16.53) | 2516(23.98) | 1368(22.70) | 932(23.11) | 1342(71.16) | 159(2.96) |
| Malaise | 43（8.67） | 37（0.56） | 3（0.16） |  |  | 2（0.18） | 3（0.07） | 87(0.82) | 3(0.05) | 83(0.79) | 7(0.12) | 7(0.17) | 7(0.37) | 3(0.06) |
| Asthenia | 67（13.51） | 647（9.72） | 42（2.27） |  |  |  | 8（0.20） | 756(7.14) | 8(0.14) | 207(1.97) | 557(9.24) | 117(2.90) | 557(29.53) | 0 |
| Headache |  | 821（12.34） |  |  |  |  | 238（5.90） | 831(7.85) | 304(5.17) | 381(3.63) | 754(12.51) | 189(4.69) | 749(39.71) | 81(1.51) |
| Dizziness | 1（0.20） | 50（0.75） |  |  |  |  | 72（1.79） | 51(0.48) | 115(1.96) | 165(1.57) | 1(0.02) | 48(1.19) | 1(0.05) | 0 |
| Increased lactate dehydrogenase | 19（3.83） | 13（0.20） |  |  |  |  | 9（0.22） | 32(0.30) | 11(0.19) | 37(0.35) | 6(0.10) | 19(0.47) | 2(0.11) | 7(0.13) |
| Increased gamma-glutamyltransferase | 17（3.43） | 87（1.31） |  |  |  |  | 249（6.17） | 19(0.18) | 253(4.31) | 272(2.59) | 0 | 29(0.72) | 0 | 70(1.30) |
| Alopecia | 109（21.98） | 669（10.05） | 590（31.86） | 42（21.54） |  | 8（0.72） | 87（2.16） | 1544(14.59) | 100(1.70) | 1635(15.59) | 9(0.15) | 627(15.55) | 9(0.48) | 0 |
| Dehydration |  | 27（0.41） |  |  | 5（8.20） |  | 12（0.30） | 32(0.30) | 24(0.41) | 56(0.53) | 0 | 24(0.60) | 0 | 0 |
| Elevated AST or ALT |  | 10（0.15） |  | 8（4.10） | 5（8.20） |  | 23（0.57） | 105(0.99) | 47(0.8) | 134(1.28) | 18(0.30) | 0 | 0 | 0 |
| Hypokalemia | 23（4.64） | 97（1.46） |  |  |  |  | 87（2.16） | 131(1.24) | 124(2.11) | 185(1.76) | 70(1.16) | 65(1.61) | 70(3.71) | 27(0.50) |
| Hyperkalemia |  | 16（0.24） |  |  |  |  | 25（0.62） | 1(0.10) | 25(0.43) | 25(0.24) | 1(0.02) | 25(0.62) | 1(0.05) | 0 |
| Hyperglycemia | 13（2.62） | 39（0.59） | 9（0.49） |  |  |  | 49（1.21） | 61(0.58) | 79(1.34) | 140(1.33) | 0 | 299(7.41) | 0 | 16(0.30) |
| Peripheral edema |  | 26（0.39） |  |  | 6（9.84） | 2（0.18） | 161（3.99） | 56(0.53) | 302(5.14) | 348(3.32) | 10(0.17) | 136(3.37) | 10(0.53) | 0 |
| Weight loss | 48（9.68） | 31（0.47） | 42（2.27） | 3（1.54） | 4（6.56） |  | 51（1.26） | 129(1.22) | 57(0.97) | 183(1.74) | 3(0.05) | 148(3.67) | 3(0.16) | 0 |
| Pyrexia | 55（11.09） | 459（6.90） | 3（0.16） | 5（2.56） |  |  | 504（12.50） | 544(5.14) | 626(10.66) | 713(6.80) | 457(7.58) | 408(10.12) | 431(22.85) | 142(2.64) |
| Infusion-related reactions |  | 5（0.08） |  | 11（5.64） |  |  | 69（1.71） | 16(0.15) | 69(1.17) | 49(0.48) | 36(0.60) | 45(1.12) | 12(0.64) | 0 |
| Musculoskeletal and connective-tissue |  | 88（1.32） |  |  |  |  |  | 88(0.83) | 0 | 88(0.84) | 0 | 0 | 0 | 0 |
