# Supplementary material for: Treatment‐related adverse events of antibody‐drug conjugates in clinical trials: A systematic review and meta‐analysis
Source: Cancer Innov. 2023 Oct 15;2(5):346–75. doi: 10.1002/cai2.97 (PMC10686142; doi:10.1002/cai2.97)
Supplement: Supplementary file 5 — eFigure 1. Incidence of treatment discontinuation and treatment‐related deaths. [file CAI2-2-346-s006.pdf]

eTable 1. The characteristics of component of ADC drugs.

ADCs drugs	Trade Name	Target	Year of initial FDA approval	Condition
Brentuximab vedotin	Adcetris	CD30	2011	Relapsed hairy leukemia and relapsed anaplastic large cell lymphoma
Ado-trastuzumab emtansine/T-DM1	Kadcyla	HER2	2013	HER2-positive metastatic breast cancer following treatment with trastuzumab and a maytansinoid
Inotuzumab ozogamicin	Besponsa	CD22	2017	Relapsed or refractory CD22-positive B-cell precursor acute lymphoblastic leukemia
Gemtuzumab ozogamicin	Mylotarg	CD33	2017	Relapsed acute myelogenous leukemia
Polatuzumab vedotin	Polivy	CD79b	2019	Relapsed or refractory diffuse large B-cell lymphoma
Fam-trastuzumab deruxtecan-nxki/ (T-DXd)/(DS-8201a)	Enhertu	HER-2	2019	Adult patients with unresectable or metastatic HER2-positive breast cancer who have received two or more prior anti-HER2 based regimens

Sacituzumab govitecan/IMMUNO-132/HRS7-SN38	TrodelvyTM	TROP-2	2020	Adult patients with metastatic triple-negative breast cancer who have received at least two prior therapies for patients with relapsed or refractory metastatic disease
Enfortumab vedotin	Padcevtm	nectin-4	2019	Adult patients with locally advanced or metastatic urothelial cancer who have received a PD-1 or PD-L1 inhibitor, and a Pt-containing therapy
Tisotumab vedotin-tftv/TF-011-MMAE/HUMAX-TF-ADC	Tivdak	TF(CD142)	2021	Recurrent or metastatic cervical cancer
Disitamab Vedotin（RC48）	Aidixi	HER-2	2018	Locally advanced or metastatic HER2 overexpressing gastric cancer (including adenocarcinoma of the gastroesophageal junction
Belantamab Mafodotin-blmf	Blenrep™	BCMA	2020	Adult patients with relapsed or refractory multiple myeloma
Moxetumomab pasudotox	Lumoxiti	CD22	2018	Adults with relapsed or refractory hairy cell leukemia
Loncastuximab tesirine-lpyl	Zynlonta	CD19	2021	Large B-cell lymphoma

RM-1929	Akalux	EGFR	2020	Recurrent head and neck squamous cell carcinoma of the head and neck that has failed 2nd line therapy

Abbreviations: ADC, Antibody-Drug Conjugates; HER2, human epidermal growth factor receptor 2; PD-1, programmed death 1; PD-L1, programmed cell death-Ligand 1

eTable 2. Search process.

Database	Keywords
**PubMed**	
#1	"Disitamab Vedotin"[Title/Abstract] OR "RC48"[Title/Abstract] OR "Tisotumab vedotin"[Title/Abstract] OR "Enfortumab vedotin"[Title/Abstract] OR "Polatuzumab vedotin"[Title/Abstract] OR "Trastuzumab Deruxtecan"[Title/Abstract] OR "(DS-8201)"[Title/Abstract] OR "Sacituzumab Govitecan"[Title/Abstract] OR "IMMU-132"[Title/Abstract] OR "Moxetumomab pasudotox"[Title/Abstract] OR "CAT-8015"[Title/Abstract] OR "HA22"[Title/Abstract] OR "Inotuzumab ozogamicin"[Title/Abstract] OR "CMC-544"[Title/Abstract] OR "Brentuximab vedotin"[Title/Abstract] OR "Trastuzumab Emtansine"[Title/Abstract] OR "T-DM1"[Title/Abstract] OR "Belantamab mafodotin"[Title/Abstract] OR "GSK2857916"[Title/Abstract] OR "Loncastuximab tesirine"[Title/Abstract] OR "ADCT-402"[Title/Abstract] OR "RM- 1929"[Title/Abstract] OR "anti-EGFR–IR700 dye conjugate"[Title/Abstract] OR "Polatuzumab vedotin"[Title/Abstract] OR "antibody-drug conjugates"[Title/Abstract] OR "ADC"[Title/Abstract]
#2	"combination"[Title/Abstract] OR "combine"[Title/Abstract] OR "combined"[Title/Abstract] OR "plus"[Title/Abstract] OR "addition"[Title/Abstract] OR "concurrent"[Title/Abstract] OR "concomitant"[Title/Abstract]
#3	"adverse events"[Title/Abstract] OR "toxicity"[Title/Abstract] OR "side effects"[Title/Abstract] OR " adverse reaction"[Title/Abstract]
#4	"cancer"[Title/Abstract] OR "neoplasm"[Title/Abstract] OR "tumor"[Title/Abstract] OR "neoplasia"[Title/Abstract] OR "malignancy"[Title/Abstract]
#5	"clinical trials as topic"[MeSH Terms] OR "clinical trial"[Publication Type] OR "phase"[Title/Abstract] OR "clinical trials"[Title/Abstract]
#6	("2001/01/01"[Date - Publication] : "2022/10/31"[Date - Publication])
#7	#1 AND #2 AND #3 AND #4 AND #5 AND #6
#8	"review"[Article type] OR "meta"[Title] OR "meta-analysis"[Title] OR "protocol"[Title]
#9	#7 NOT #8
**EMBASE**	
#1	(Disitamab Vedotin OR RC48 OR Tisotumab vedotin OR Enfortumab vedotin OR Polatuzumab vedotin OR Trastuzumab Deruxtecan OR DS-8201 OR Sacituzumab Govitecan OR IMMU-132 OR Moxetumomab pasudotox OR CAT-8015 OR HA22 OR Inotuzumab ozogamicin OR CMC-544 OR Brentuximab vedotin OR Trastuzumab Emtansine OR T-DM1 OR Belantamab mafodotin OR GSK2857916 OR Loncastuximab tesirine OR ADCT-402 OR RM- 1929 OR anti-EGFR–IR700 dye conjugate OR Polatuzumab vedotin OR antibody-drug conjugates OR ADC):ti,ab,kw
#2	(combination OR combine OR combined OR plus OR addition OR concurrent OR concomitant):ti,ab,kw
#3	trial/exp OR 'clinical trials'/exp OR 'phase 1 clinical trial'/exp OR 'phase 2 clinical trial'/exp OR 'phase 3 clinical trial'/exp
#4	[article]/lim OR [article in press]/lim
#5	[humans]/lim
#6	#1 AND #2 AND #3 AND #4 AND #5
**CENTRAL**	
#1	(Disitamab Vedotin OR RC48 OR Tisotumab vedotin OR Enfortumab vedotin OR Polatuzumab vedotin OR Trastuzumab Deruxtecan OR DS-8201 OR Sacituzumab Govitecan OR IMMU-132 OR Moxetumomab pasudotox OR CAT-8015 OR HA22 OR Inotuzumab ozogamicin OR CMC-544 OR Brentuximab vedotin

	OR Trastuzumab Emtansine OR T-DM1 OR Belantamab mafodotin OR GSK2857916 OR Loncastuximab tesirine OR ADCT-402 OR RM- 1929 OR anti-EGFR–IR700 dye conjugate OR Polatuzumab vedotin OR antibody-drug conjugates OR ADC)
#2	(combination OR combine OR combined OR plus OR addition OR concurrent OR concomitant):ti,ab,kw
#3	(‘clinical trials as topic' OR trial):ti,ab,kw
#4	("conference" OR "review"):pt
#5	#1 AND #2 AND #3 NOT #4
#6	Publication date: Between Jan 2001 and October 2022

eTable 3. Component of Antibody-Drug Conjugates.

Antibody-Drug Conjugates	Target	Linker	Payload
Disitamab Vedotin (RC48)	HER-2	Cleavable	MMAE
Tisotumab vedotin	TF(CD142)	Cleavable	MMAE
Enfortumab vedotin	Nectin-4	Cleavable	MMAE
Polatuzumab vedotin	CD79b	Cleavable	MMAE
Trastuzumab Deruxtecan (DS-8201)	HER-2	Cleavable	Deruxtecan
Sacituzumab Govitecan (IMMU-132)	TROP-2	Cleavable	SN-38
Moxetumomab pasudotox (CAT-8015, HA22)	CD22	Cleavable	MMAE
Inotuzumab ozogamicin (CMC-544)	CD22	Cleavable	Calicheamicin
Brentuximab vedotin	CD30	Cleavable	MMAE
Trastuzumab Emtansine(T-DM1)	HER2	Non-cleavable	DM1
Belantamab mafodotin (GSK2857916)	BCMA	Non-cleavable	MMAF
Loncastuximab tesirine (ADCT-402)	CD19	Cleavable	PBD dimer SG3199
RM- 1929 (anti-EGFR–IR700 dye conjugate)	EGFR	Cleavable	IRDye700DX
Polatuzumab vedotin	CD79b	Cleavable	MMAE

eTable 4. Incidence and types of treatment-related according to cancer type and component.

Variables, no. (%)	Cancer type							Component						
	Gastrointestinal cancer (n =496)	Breast cancer (n =6,655)	Urothelial carcinoma (n =1,852)	NSCLC or colorectal cancer or other solid cancer (n =195)	Head and Neck Squamous Cell Carcinoma (n = 61)	Uterine Cancer (n=1,108)	Hematological cancer (n=4,033)	Antibody		Linker		payload		
								For Soli（n=10,585）	For Hematological hematological malignancies（n=5,875）	Cleavable (n =10,488)	Non-cleavable (n =6,026)	DMEM(n=4,033)	Deruxtecan（Dxd)/Camptothecin(n=1,886)	DM1 (n=5,380)
**Deaths**	**7（1.41）**	**23（0.35）**	**12（0.65）**	**5（2.56）**	**0**	**1（0.09）**	**81（2.01）**	**53(0.50)**	**89(1.51)**	**126(1.20)**	**16(0.27)**	**23(0.57)**	**65(3.45)**	**11(0.20)**
**Respiratory**	**28（5.65）**	**3861（58.01）**	**1（0.05）**	**30(15.38)**	**183(300)**		**216(5.36)**	**901（8.51）**	**561(9.55)**	**801(7.64)**	**661（10.97）**	**255(6.32)**	**633（33.56）**	**67（1.23）**
Interstitial lung disease	28（5.65）	174（2.61）	1（0.05）	27（13.85）	4（6.56）		45（1.12）	206(1.95)	55(0.94)	165(1.57)	96(1.59)	16(0.40)	68(3.61)	15(0.28)
Dyspnoea		348（5.23）		3（1.54）			171（4.24）	352(3.33)	279(4.75)	335(3.20)	296(4.91)	118(2.93)	296(15.69)	40(0.74)
Productive cough		3339（50.17）			179（293.44）			343(3.24)	227(3.86)	301(2.87)	269(4.46)	121(3.00)	269(14.26)	12(0.22)

**Gastrointestinal**	**536（108.06）**	**6360（95.57）**	**1170（63.17）**	200(102.56)	**6（9.84）**	**850(76.71)**	**1734(43.00)**	**8910（84.18）**	**2304(39.22)**	**7824(74.60)**	**3355（55.68）**	**2343（58.09）**	**3285（227.20）**	**371（6.90）**
Decreased appetite	133（26.81）	730（10.97）	241（13.01）	34（17.44）		2（0.18）	188（4.66）	1239(11.71)	270(4.60)	1134(10.81)	375(6.22)	467(11.58)	370(19.62)	20(0.37)
Nausea	137（27.62）	2440（36.66）	507（27.38）	79（40.51）		833（75.18）	605（15）	3276(30.95)	765(13.02)	2604(24.83)	1402(23.27)	769(19.07)	1353(71.74)	154(2.86)
Diarrhea	71（14.31）	1034（15.54）	191（10.31）	29（14.81）			380（9.42）	1699(16.05)	476(8.10)	1690(16.11)	485(8.05)	563(13.95)	469(24.87	42(0.78)
Vomiting	80（16.13）	1035（15.55）	231（12.47）	36（18.46）			257（6.37）	1399(13.22)	365(6.21)	1295(12.35)	469(7.78)	212(5.27)	469(24.87)	93(1.73)
Constipation	67（13.51）	969（14.56）		20（10.26）	6（9.84）	0	166（4.12）	1096(10.35)	267(4.54)	772(7.36)	591(9.81)	177(4.39)	591(31.34)	43(0.80)
Abdominal pain	48（9.68）	133（2.00）				15（1.35）	113（2.80）	180(1.70)	136(2.31)	288(2.75)	28(0.46)	138(3.42)	28(1.48)	11(0.20)
Anorexia		19（0.29）		2（1.03）			25（0.62）	21(0.20)	25(0.43)	41(0.39)	5(0.08)	17(0.42)	5(0.27)	8(0.15)
**Cardiovascular**		102（1.53）			0		95(2.36)	110（1.04）	78(1.33)	78(0.74)	110（1.83）	81（2.00）	110（5.83）	5（0.09）

Uncontrolled hypertension		97（1.46）					28（0.69）	105(0.99)	28(0.48)	28(0.27)	105(1.74)	28(0.69)	105(5.57)	0
Hypotension		5（0.08）					31（0.77）	5(0.05)	40(0.68)	40(0.38)	5(0.08)	17(0.42)	5(0.27)	5(0.09)
Capillary leak syndrome							26（0.64）	0	0	0	0	26(0.64)	0	0
Tachycardia							10（0.25）	0	10(0.17)	10(0.10)	0	10(0.25)	0	0
**Neurologic**	**7（1.41）**	**280（4.21）**	**336（18.14）**		**9（14.75）**	**3（0.27）**	**526(13.04)**	**836（2.23）**	**397(6.76)**	**1089(10.38)**	**144（2.39）**	**1097（27.20）**	**144（7.64）**	**0**
Peripheral sensory neuropathy		141（2.12）	282（15.23）		9（14.75）	3（0.27）	449（11.13）	501(4.73)	273(4.65)	633(6.04)	141(2.34)	725(17.98)	141(7.48)	0
Peripheral motor neuropathy	7（1.41）	52（0.78）					67（1.66）	0	74(1.26)	74(0.71)	0	74(1.83)	0	0
Hypoaesthesia		1（0.02）	54（2.92）				1（0.02）	249(2.35)	41(0.70)	289(2.18)	1(0.02)	289(7.17)	1(0.05)	0
Neuropathy		86（1.29）					9（0.22）	86(0.23)	9(0.15)	93(0.89)	2(0.03)	9(0.22)	2(0.11)	0
**Hematolog**	**641**	**3313**	**535(28.89)**	105(53.85)	**10(16.39)**	**226(20.40**	**1836(45.52)**	**4860**	**3997(68.**	**5958(5**	**1584**	**1578**	**1288**	**779**

**ic**	**（129.23）**	**（49.78）**				**)**		**（45.91）**	**03)**	**6.81)**	**（26.29）**	**（39.13）**	**（68.29）**	**（14.48）**
Leukopenia	165（33.27）	464（6.97）	44（2.38）	21（10.80）			103（2.55）	837(7.91)	192(3.27)	994(9.48)	35(0.58)	375(9.30)	35(1.86)	87(1.62)
Decreased neutrophil count	187（37.70）	864（12.98）	169（9.13）	32（16.41）		3（0.27）	593（14.71）	1315(12.42)	2174(37.00)	2040(19.45)	134(2.22)	572(14.18)	92(4.88)	183(3.40)
Febrile neutropenia		31（0.47）	40（2.16）			27（2.44）	93（2.31）	71(0.67)	137(2.33)	199(1.90)	9(0.15)	18(0.45)	9(0.48)	108(2.01)
Anemia	169（34.07）	889（13.36）	261（14.09）	32（16.41）	10（16.39）	196（17.69）	374（9.27）	1407(13.29)	545(9.28)	1512(14.42)	440(7.30)	273(6.77)	335(17.76)	118(2.19)
Decreased platelet count	79（15.93）	1007（15.13）	9（0.49）	20（10.26）			542（13.44）	1119(10.57)	840(14.30)	1038(9.90)	921(15.28)	293(7.27)	785(41.62)	220(4.19)
Decreased lymphocyte count	41（8.27）	58（0.87）	12（0.65）				131（3.25）	111(1.05)	109(1.86)	175(1.67)	45(0.75)	47(1.17)	32(1.70)	63(1.17)
**Urinary**	**5（1.01）**	**205（3.08）**	**9(0.49)**			**11(0.99)**	**16(0.40)**	**233（2.20）**	**16(0.27)**	**85(0.81)**	**164（2.72）**	**38（0.94）**	**164（8.70）**	**0**
Urinary tract		202（3.04）	9（0.49）			11（0.99）		222(2.10)	0	58(0.55)	164（2.72	11(0.27)	164(8.70)	0

infection											）			
Cystitis		3（0.05）						0	0	0	0	0	0	0
Protein present in urine	5（1.01）							11(0.10)	0	11(0.10)	0	11(0.27)	0	0
Hemolytic uremic syndrome							16（0.40）	0	16(0.27)	16(0.15)	0	16(0.40)	0	0
**Ear, Nose and Throat System**	**0**	**653（9.81）**	**57(3.08)**	6（3.08）	**4（6.56）**		**63(1.56)**	**882（8.33)**	**82(1.40)**	**317(3.02)**	**647（10.67）**	**114（2.83）**	**643（34.09）**	**4（0.07）**
Oropharyngeal pain		2（0.03）			4（6.56）		20（0.50）	6(0.06)	20(0.34)	24(0.23)	2(0.03)	20(0.50)	2(0.11)	0
Rhinitis							24（0.60）	0	0	0	0	0	0	0
Epistaxis		635（9.54）		6（3.08）			4（0.10）	677(6.40)	4(0.07)	40(0.38)	641(10.64)	36(0.89)	641(33.98)	4(0.07)
Nasopharyngitis							15（0.37）	0	58(0.99)	58(0.55)	0	58(1.44)	0	0
Dysgeusia		16（0.24）	57（3.08）					199(1.88)	0	195(1.86)	4(0.07)	0	0	0
**Ophthalmic**	**0**	**3（0.05）**	**0**				**300(7.44)**	**17(0.16)**	**324(5.51)**	**20(0.19)**	**321（5.33）**	**20（0.50）**	**3（0.16）**	**0**
Dry eye		3（0.05）					20（0.50）	16(0.	20(0.34)	13(0.12	23(0.3	13(0.	3(0.16)	0

								15)		)	8)	32)		
Corneal lesion							190（4.71）	0	214(3.64)	0	214(3.55)	0	0	0
Vision blurred							39（0.97）	1(0.01)	39(0.66)	7(0.07)	33(0.53)	7(0.17)	0	0
Change in BCVA							51（1.26）	0	51(0.87)	0	51(0.85)	0	0	0
**Infection**	**0**	**5（0.08）**					**21(0.52)**	**86(0.81)**	**137(2.33)**	**223(2.13)**	**0**	**3（0.07）**	**0**	**7（0.13）**
Cellulitis		5（0.08）						0	0	0	0	0	0	0
Sepsis							10（0.25）	0	10(0.17)	10(0.10)	0	3(0.07)	0	7(0.13)
Septic shock							6（0.15）	0	0	0	0	0	0	0
infection							5（0.12）	86(0.81)	127(2.16)	213(2.03)	0			
**Dermatological**	**41（8.27）**	**97（1.46）**	**235(12.69)**	3（1.54）	**5（8.20）**	**0**	**387(9.60)**	**528(4.98)**	**543(9.24)**	**1058(10.09)**	**13（0.22）**	**639（15.84）**	**13（0.69）**	**19（0.35）**
Skin rash	5（1.01）	65（0.98）	10（0.54）	3（1.54）	5（8.20）		182（4.51）	106(1.00)	261(4.44)	360(3.43)	7(0.12)	120(2.98)	7(0.37)	19(0.35)
Maculopapular rash			83（4.48）				59（1.46）	116(1.10)	91(1.55)	207(1.97)	0	126(3.12)	0	0
Dry skin		15（0.23）	34（1.84）					77(0.73)	0	77(0.73)	0	60(1.49)	0	0

Pruritus	36（7.26）	17（0.26）	108（5.83）				146（3.62）	229(2.16)	191(3.25)	414(3.95)	6(0.10)	333(8.26)	6(0.32)	0
**Others**	**557（112.30）**	**5787（86.96）**	**1243(67.12)**	123（63.08）	**20(32.79)**	**279(25.18)**	**2652(65.76)**	**8053(76.08)**	**3324(56.58)**	**7550(71.99)**	**3852（63.92）**	**3393（84.13）**	**3749（198.78）**	**514（9.55）**
Back pain	16（3.23）	59（0.89）					86（2.13）	40(0.38)	52(0.89)	98(0.93)	19(0.32)	84(2.08)	19(1.01)	0
Joint pain	15（3.02）	539（8.10）					126（3.12）	560(5.29)	126(2.14)	175(1.67)	511(8.48)	138(3.42)	511(27.09)	9(9.55)
Physical pain		36（0.54）					28（0.69）	57(0.54)	28(0.48)	60(0.57)	25(0.41)	53(1.31)	25(1.33)	0
Fatigue	131（26.41）	2031（30.52）	554（29.91）	54（27.69）		267（24.1）	765（18.97）	2913(27.52)	971(16.53)	2516(23.98)	1368(22.70)	932(23.11)	1342(71.16)	159(2.96)
Malaise	43（8.67）	37（0.56）	3（0.16）			2（0.18）	3（0.07）	87(0.82)	3(0.05)	83(0.79)	7(0.12)	7(0.17)	7(0.37)	3(0.06)
Asthenia	67（13.51）	647（9.72）	42（2.27）				8（0.20）	756(7.14)	8(0.14)	207(1.97)	557(9.24)	117(2.90)	557(29.53)	0
Headache		821（12.34）					238（5.90）	831(7.85)	304(5.17)	381(3.63)	754(12.51)	189(4.69)	749(39.71)	81(1.51)
Dizziness	1（0.20）	50（0.75）					72（1.79）	51(0.48)	115(1.96)	165(1.57)	1(0.02)	48(1.19)	1(0.05)	0
Increased lactate dehydrogenase	19（3.83）	13（0.20）					9（0.22）	32(0.30)	11(0.19)	37(0.35)	6(0.10)	19(0.47)	2(0.11)	7(0.13)

Increased gamma-glutamyltransferase	17（3.43）	87（1.31）					249（6.17）	19(0.18)	253(4.31)	272(2.59)	0	29(0.72)	0	70(1.30)
Alopecia	109（21.98）	669（10.05）	590（31.86）	42（21.54）		8（0.72）	87（2.16）	1544(14.59)	100(1.70)	1635(15.59)	9(0.15)	627(15.55)	9(0.48)	0
Dehydration		27（0.41）			5（8.20）		12（0.30）	32(0.30)	24(0.41)	56(0.53)	0	24(0.60)	0	0
Elevated AST or ALT		10（0.15）		8（4.10）	5（8.20）		23（0.57）	105(0.99)	47(0.8)	134(1.28)	18(0.30)	0	0	0
Hypokalemia	23（4.64）	97（1.46）					87（2.16）	131(1.24)	124(2.11)	185(1.76)	70(1.16)	65(1.61)	70(3.71)	27(0.50)
Hyperkalemia		16（0.24）					25（0.62）	1(0.10)	25(0.43)	25(0.24)	1(0.02)	25(0.62)	1(0.05)	0
Hyperglycemia	13（2.62）	39（0.59）	9（0.49）				49（1.21）	61(0.58)	79(1.34)	140(1.33)	0	299(7.41)	0	16(0.30)
Peripheral edema		26（0.39）			6（9.84）	2（0.18）	161（3.99）	56(0.53)	302(5.14)	348(3.32)	10(0.17)	136(3.37)	10(0.53)	0
Weight loss	48（9.68）	31（0.47）	42（2.27）	3（1.54）	4（6.56）		51（1.26）	129(1.22)	57(0.97)	183(1.74)	3(0.05)	148(3.67)	3(0.16)	0
Pyrexia	55（11.09）	459（6.90）	3（0.16）	5（2.56）			504（12.50）	544(5.14)	626(10.66)	713(6.80)	457(7.58)	408(10.12)	431(22.85)	142(2.64)
Infusion-		5（0.08）		11（5.64）			69（1.71）	16(0.	69(1.17)	49(0.48	36(0.6	45(1.	12(0.64)	0

related reactions								15)		)	0)	12)		
Musculo skeletal and connective-tissue		88（1.32）						88(0.83)	0	88(0.84)	0	0	0	0

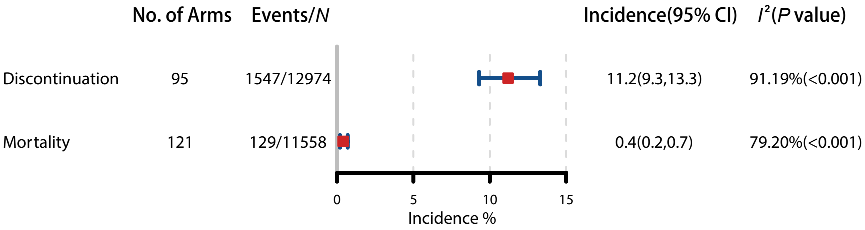

No. of Arms
Events/N
Incidence(95% CI)
I²(P value)
Discontinuation
95
1547/12974
11.2(9.3,13.3)
91.19%(<0.001)
Mortality
121
129/11558
0.4(0.2,0.7)
79.20%(<0.001)
0
5
10
15
Incidence %

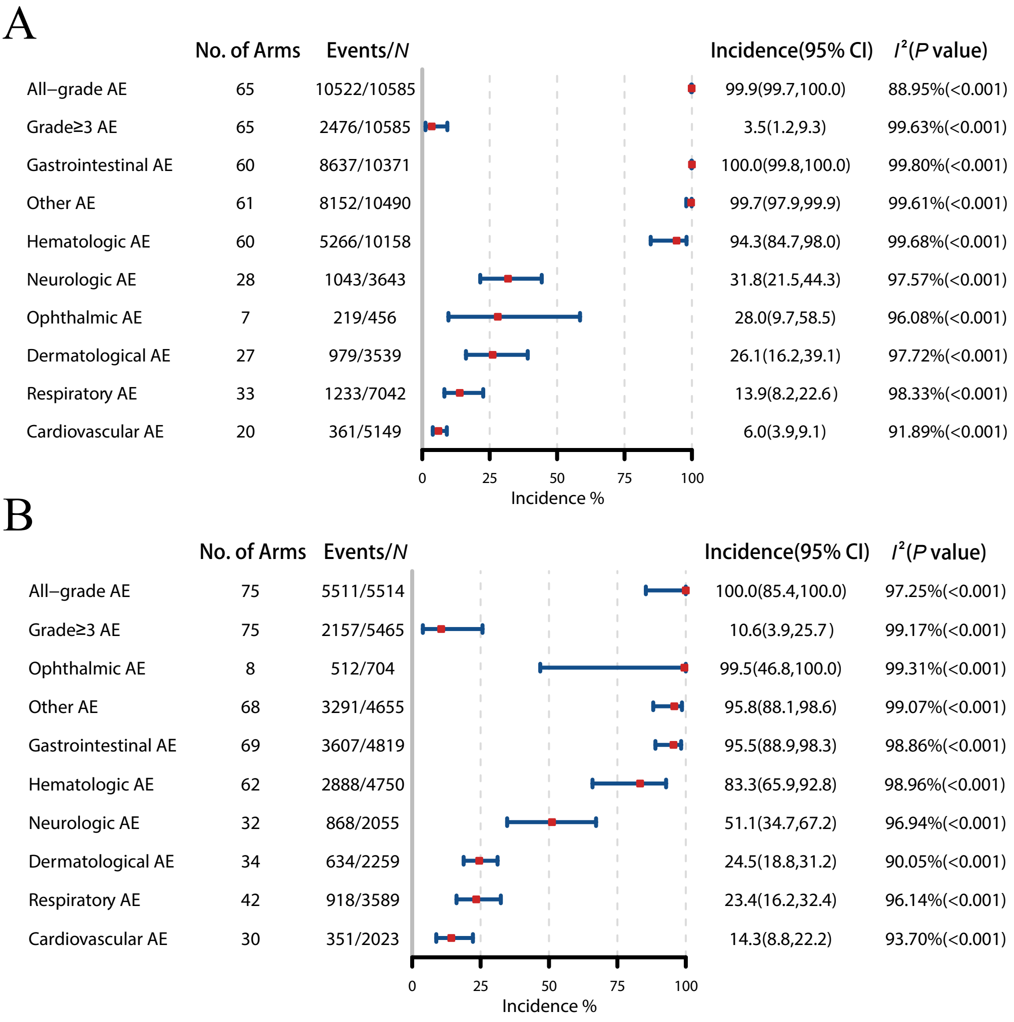
A
No. of Arms
Events/N
Incidence(95% CI)
I²(P value)
All–grade AE 65 10522/10585 99.9(99.7,100.0) 88.95%(<0.001)
Grade≥3 AE 65 2476/10585 3.5(1.2,9.3) 99.63%(<0.001)
Gastrointestinal AE 60 8637/10371 100.0(99.8,100.0) 99.80%(<0.001)
Other AE 61 8152/10490 99.7(97.9,99.9) 99.61%(<0.001)
Hematologic AE 60 5266/10158 94.3(84.7,98.0) 99.68%(<0.001)
Neurologic AE 28 1043/3643 31.8(21.5,44.3) 97.57%(<0.001)
Ophthalmic AE 7 219/456 28.0(9.7,58.5) 96.08%(<0.001)
Dermatological AE 27 979/3539 26.1(16.2,39.1) 97.72%(<0.001)
Respiratory AE 33 1233/7042 13.9(8.2,22.6) 98.33%(<0.001)
Cardiovascular AE 20 361/5149 6.0(3.9,9.1) 91.89%(<0.001)
0 25 50 75 100
Incidence %
B
No. of Arms
Events/N
Incidence(95% CI)
I²(P value)
All–grade AE 75 5511/5514 100.0(85.4,100.0) 97.25%(<0.001)
Grade≥3 AE 75 2157/5465 10.6(3.9,25.7) 99.17%(<0.001)
Ophthalmic AE 8 512/704 99.5(46.8,100.0) 99.31%(<0.001)
Other AE 68 3291/4655 95.8(88.1,98.6) 99.07%(<0.001)
Gastrointestinal AE 69 3607/4819 95.5(88.9,98.3) 98.86%(<0.001)
Hematologic AE 62 2888/4750 83.3(65.9,92.8) 98.96%(<0.001)
Neurologic AE 32 868/2055 51.1(34.7,67.2) 96.94%(<0.001)
Dermatological AE 34 634/2259 24.5(18.8,31.2) 90.05%(<0.001)
Respiratory AE 42 918/3589 23.4(16.2,32.4) 96.14%(<0.001)
Cardiovascular AE 30 351/2023 14.3(8.8,22.2) 93.70%(<0.001)
0 25 50 75 100
Incidence %

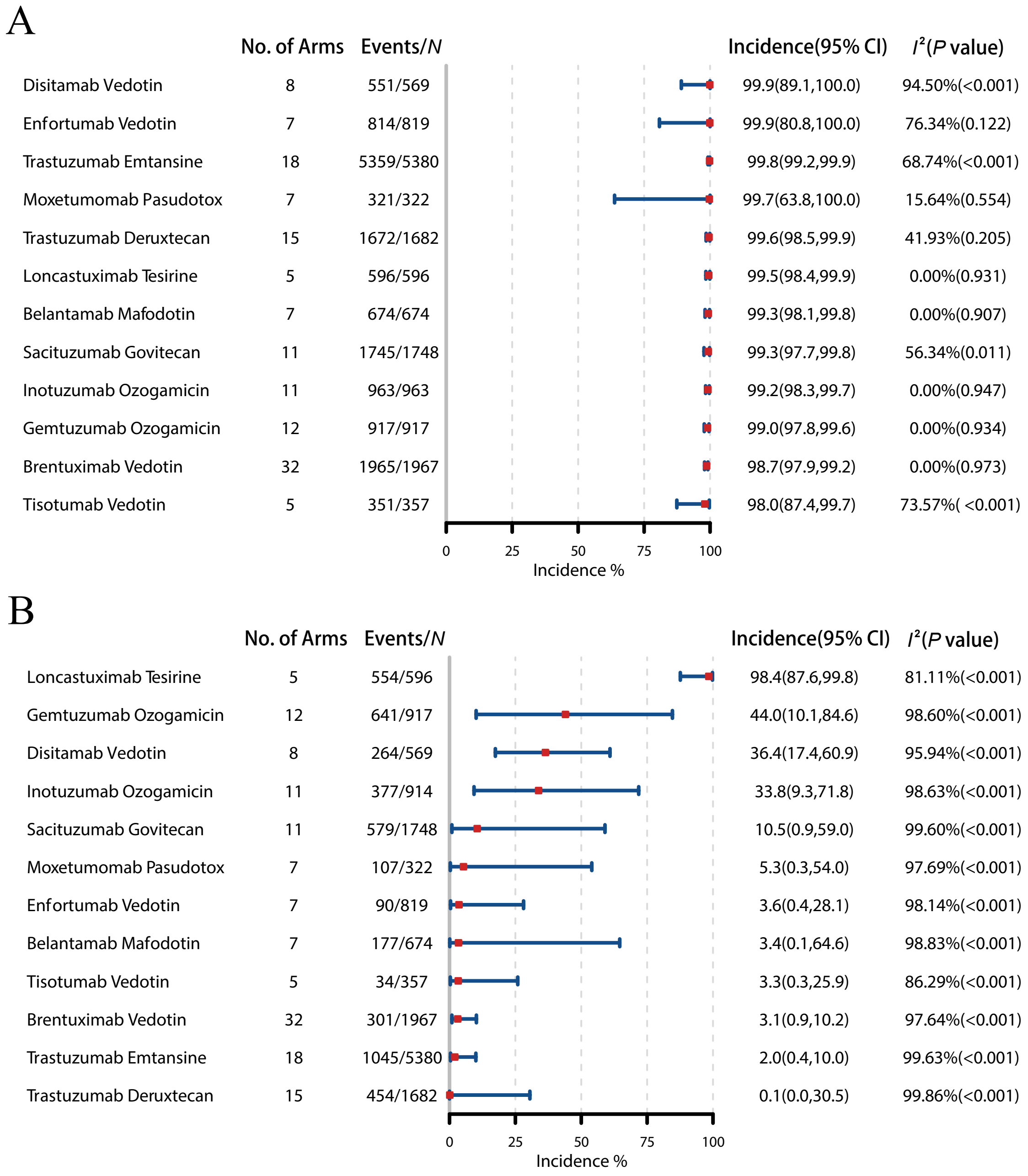
A
No. of Arms
Events/N
Incidence(95% CI)
I²(P value)
Disitamab Vedotin 8 551/569 99.9(89.1,100.0) 94.50%(<0.001)
Enfortumab Vedotin 7 814/819 99.9(80.8,100.0) 76.34%(0.122)
Trastuzumab Emtansine 18 5359/5380 99.8(99.2,99.9) 68.74%(<0.001)
Moxetumomab Pasudotox 7 321/322 99.7(63.8,100.0) 15.64%(0.554)
Trastuzumab Deruxtecan 15 1672/1682 99.6(98.5,99.9) 41.93%(0.205)
Loncastuximab Tesirine 5 596/596 99.5(98.4,99.9) 0.00%(0.931)
Belantamab Mafodotin 7 674/674 99.3(98.1,99.8) 0.00%(0.907)
Sacituzumab Govitecan 11 1745/1748 99.3(97.7,99.8) 56.34%(0.011)
Inotuzumab Ozogamicin 11 963/963 99.2(98.3,99.7) 0.00%(0.947)
Gemtuzumab Ozogamicin 12 917/917 99.0(97.8,99.6) 0.00%(0.934)
Brentuximab Vedotin 32 1965/1967 98.7(97.9,99.2) 0.00%(0.973)
Tisotumab Vedotin 5 351/357 98.0(87.4,99.7) 73.57%(<0.001)
0 25 50 75 100
Incidence %
B
No. of Arms
Events/N
Incidence(95% CI)
I²(P value)
Loncastuximab Tesirine 5 554/596 98.4(87.6,99.8) 81.11%(<0.001)
Gemtuzumab Ozogamicin 12 641/917 44.0(10.1,84.6) 98.60%(<0.001)
Disitamab Vedotin 8 264/569 36.4(17.4,60.9) 95.94%(<0.001)
Inotuzumab Ozogamicin 11 377/914 33.8(9.3,71.8) 98.63%(<0.001)
Sacituzumab Govitecan 11 579/1748 10.5(0.9,59.0) 99.60%(<0.001)
Moxetumomab Pasudotox 7 107/322 5.3(0.3,54.0) 97.69%(<0.001)
Enfortumab Vedotin 7 90/819 3.6(0.4,28.1) 98.14%(<0.001)
Belantamab Mafodotin 7 177/674 3.4(0.1,64.6) 98.83%(<0.001)
Tisotumab Vedotin 5 34/357 3.3(0.3,25.9) 86.29%(<0.001)
Brentuximab Vedotin 32 301/1967 3.1(0.9,10.2) 97.64%(<0.001)
Trastuzumab Emtansine 18 1045/5380 2.0(0.4,10.0) 99.63%(<0.001)
Trastuzumab Deruxtecan 15 454/1682 0.1(0.0,30.5) 99.86%(<0.001)
0 25 50 75 100
Incidence %

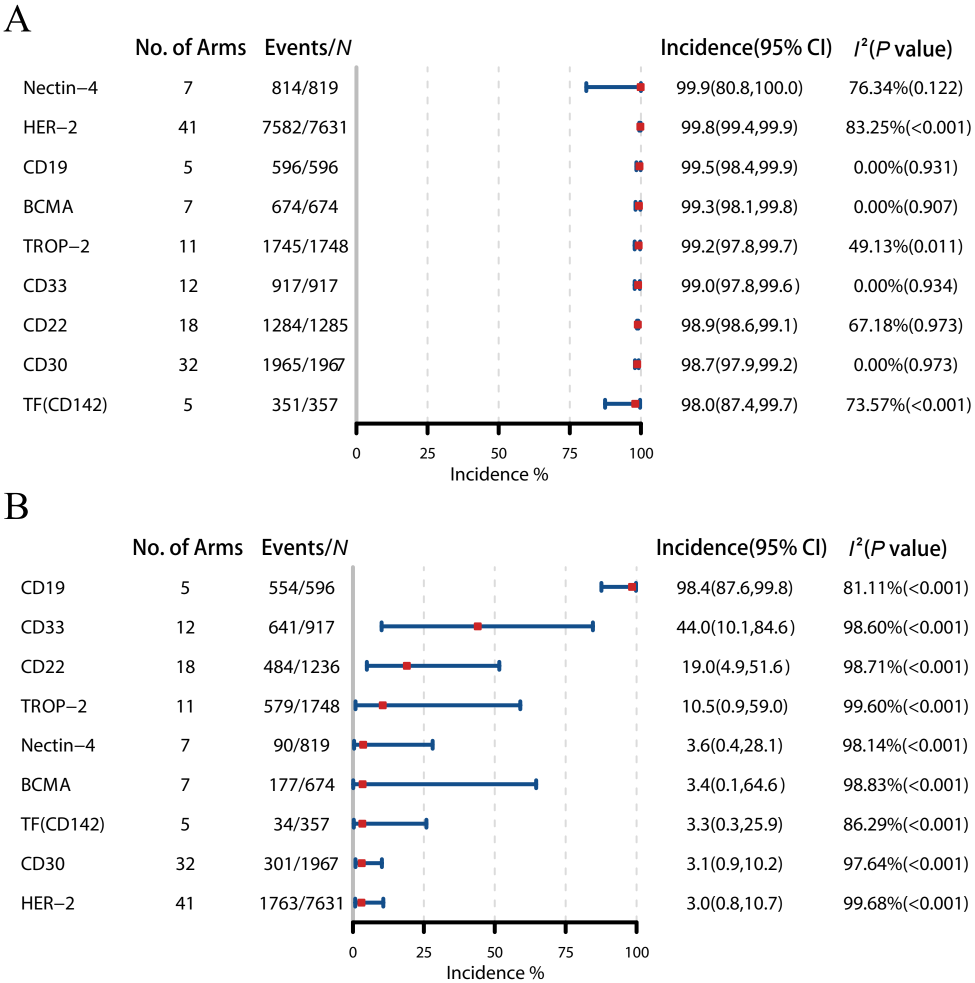

A
No. of Arms
Events/N
Incidence(95% CI)
I²(P value)
Nectin–4 7 814/819 99.9(80.8,100.0) 76.34%(0.122)
HER–2 41 7582/7631 99.8(99.4,99.9) 83.25%(<0.001)
CD19 5 596/596 99.5(98.4,99.9) 0.00%(0.931)
BCMA 7 674/674 99.3(98.1,99.8) 0.00%(0.907)
TROP–2 11 1745/1748 99.2(97.8,99.7) 49.13%(0.011)
CD33 12 917/917 99.0(97.8,99.6) 0.00%(0.934)
CD22 18 1284/1285 98.9(98.6,99.1) 67.18%(0.973)
CD30 32 1965/1967 98.7(97.9,99.2) 0.00%(0.973)
TF(CD142) 5 351/357 98.0(87.4,99.7) 73.57%(<0.001)
0
25
50
75
100
Incidence %
B
No. of Arms
Events/N
Incidence(95% CI)
I²(P value)
CD19 5 554/596 98.4(87.6,99.8) 81.11%(<0.001)
CD33 12 641/917 44.0(10.1,84.6) 98.60%(<0.001)
CD22 18 484/1236 19.0(4.9,51.6) 98.71%(<0.001)
TROP–2 11 579/1748 10.5(0.9,59.0) 99.60%(<0.001)
Nectin–4 7 90/819 3.6(0.4,28.1) 98.14%(<0.001)
BCMA 7 177/674 3.4(0.1,64.6) 98.83%(<0.001)
TF(CD142) 5 34/357 3.3(0.3,25.9) 86.29%(<0.001)
CD30 32 301/1967 3.1(0.9,10.2) 97.64%(<0.001)
HER–2 41 1763/7631 3.0(0.8,10.7) 99.68%(<0.001)
0
25
50
75
100
Incidence %

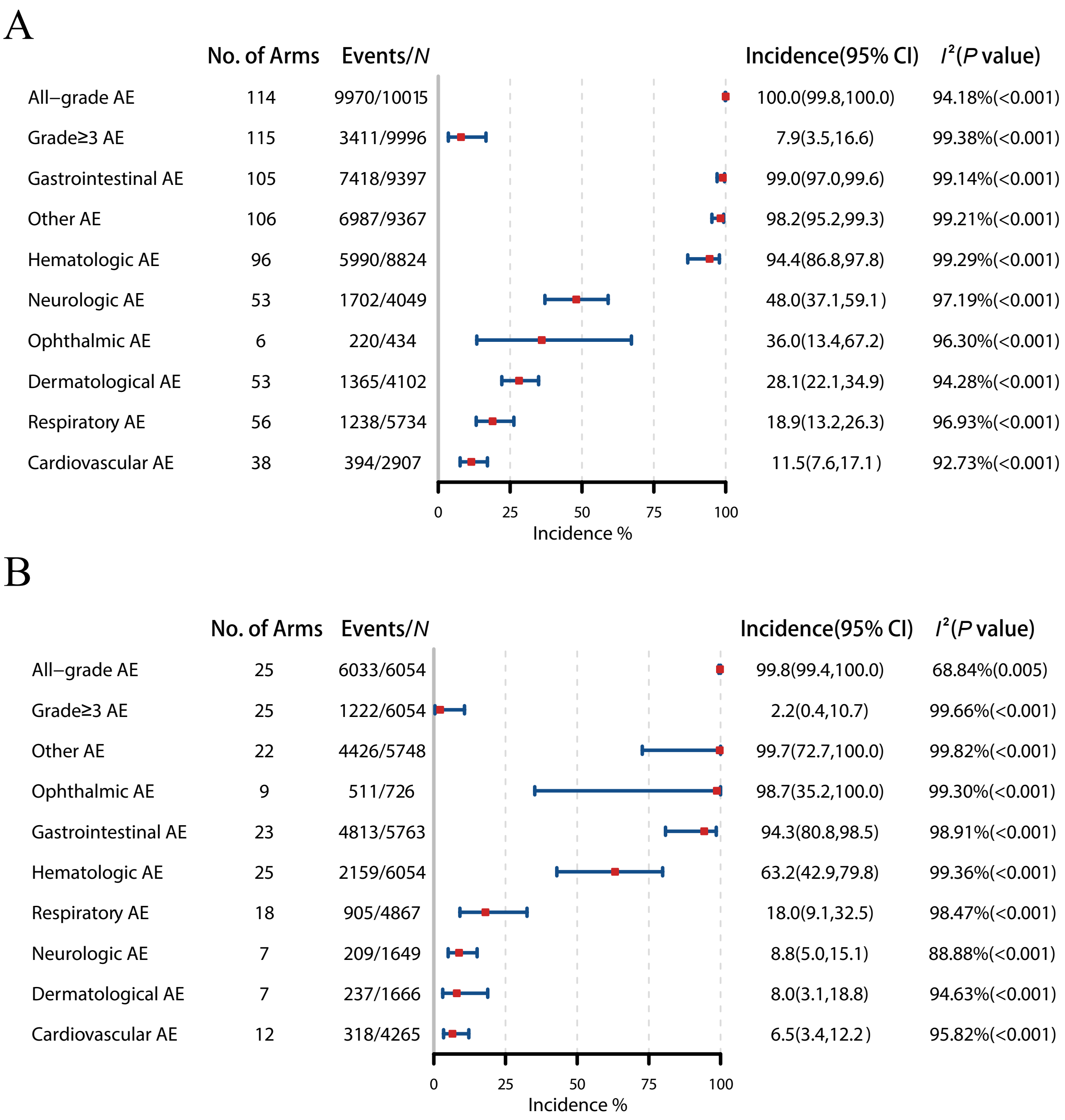

A
No. of Arms
Events/N
Incidence(95% CI)
I²(P value)
All−grade AE 114 9970/10015 100.0(99.8,100.0) 94.18%(<0.001)
Grade≥3 AE 115 3411/9996 7.9(3.5,16.6) 99.38%(<0.001)
Gastrointestinal AE 105 7418/9397 99.0(97.0,99.6) 99.14%(<0.001)
Other AE 106 6987/9367 98.2(95.2,99.3) 99.21%(<0.001)
Hematologic AE 96 5990/8824 94.4(86.8,97.8) 99.29%(<0.001)
Neurologic AE 53 1702/4049 48.0(37.1,59.1) 97.19%(<0.001)
Ophthalmic AE 6 220/434 36.0(13.4,67.2) 96.30%(<0.001)
Dermatological AE 53 1365/4102 28.1(22.1,34.9) 94.28%(<0.001)
Respiratory AE 56 1238/5734 18.9(13.2,26.3) 96.93%(<0.001)
Cardiovascular AE 38 394/2907 11.5(7.6,17.1) 92.73%(<0.001)
0
25
50
75
100
Incidence %
B
No. of Arms
Events/N
Incidence(95% CI)
I²(P value)
All−grade AE 25 6033/6054 99.8(99.4,100.0) 68.84%(0.005)
Grade≥3 AE 25 1222/6054 2.2(0.4,10.7) 99.66%(<0.001)
Other AE 22 4426/5748 99.7(72.7,100.0) 99.82%(<0.001)
Ophthalmic AE 9 511/726 98.7(35.2,100.0) 99.30%(<0.001)
Gastrointestinal AE 23 4813/5763 94.3(80.8,98.5) 98.91%(<0.001)
Hematologic AE 25 2159/6054 63.2(42.9,79.8) 99.36%(<0.001)
Respiratory AE 18 905/4867 18.0(9.1,32.5) 98.47%(<0.001)
Neurologic AE 7 209/1649 8.8(5.0,15.1) 88.88%(<0.001)
Dermatological AE 7 237/1666 8.0(3.1,18.8) 94.63%(<0.001)
Cardiovascular AE 12 318/4265 6.5(3.4,12.2) 95.82%(<0.001)
0
25
50
75
100
Incidence %

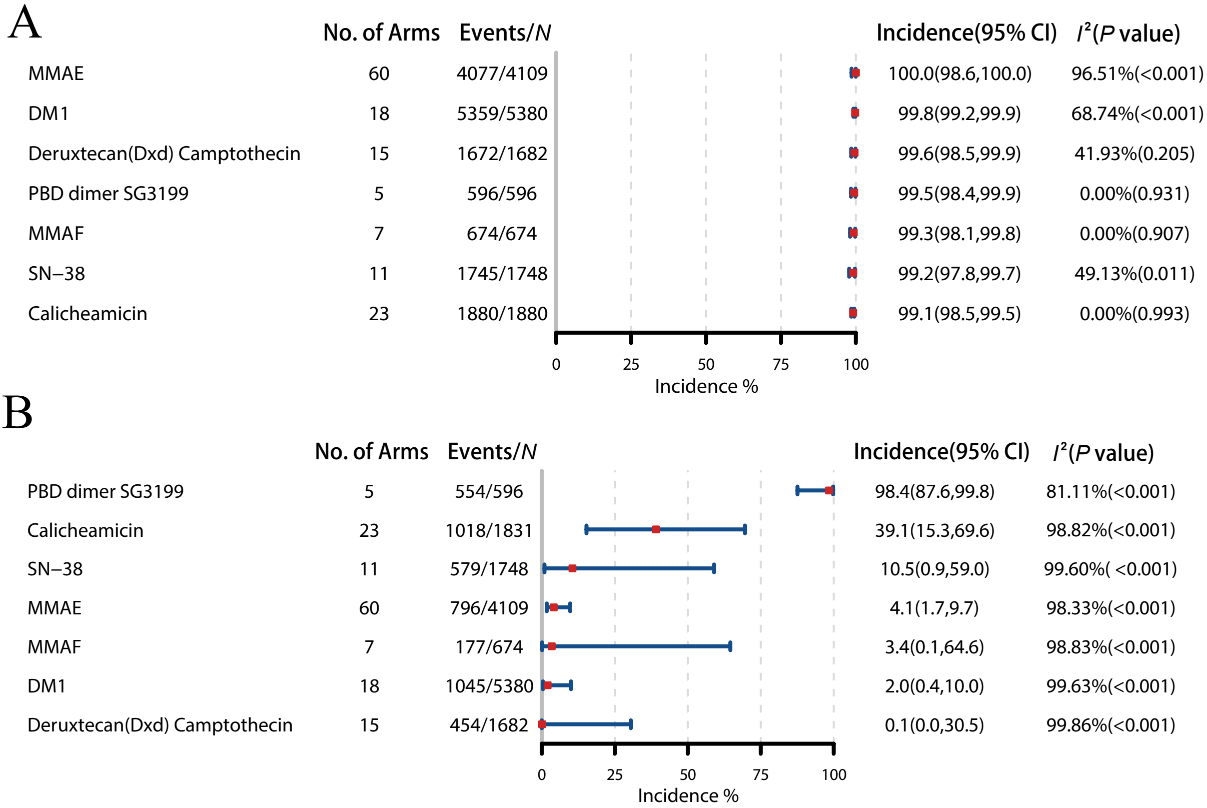
A
No. of Arms
Events/N
Incidence(95% CI)
I²(P value)
MMAE 60 4077/4109 100.0(98.6,100.0) 96.51%(<0.001)
DM1 18 5359/5380 99.8(99.2,99.9) 68.74%(<0.001)
Deruxtecan(Dxd) Camptothecin 15 1672/1682 99.6(98.5,99.9) 41.93%(0.205)
PBD dimer SG3199 5 596/596 99.5(98.4,99.9) 0.00%(0.931)
MMAF 7 674/674 99.3(98.1,99.8) 0.00%(0.907)
SN–38 11 1745/1748 99.2(97.8,99.7) 49.13%(0.011)
Calicheamicin 23 1880/1880 99.1(98.5,99.5) 0.00%(0.993)
0
25
50
75
100
Incidence %
B
No. of Arms
Events/N
Incidence(95% CI)
I²(P value)
PBD dimer SG3199 5 554/596 98.4(87.6,99.8) 81.11%(<0.001)
Calicheamicin 23 1018/1831 39.1(15.3,69.6) 98.82%(<0.001)
SN–38 11 579/1748 10.5(0.9,59.0) 99.60%(<0.001)
MMAE 60 796/4109 4.1(1.7,9.7) 98.33%(<0.001)
MMAF 7 177/674 3.4(0.1,64.6) 98.83%(<0.001)
DM1 18 1045/5380 2.0(0.4,10.0) 99.63%(<0.001)
Deruxtecan(Dxd) Camptothecin 15 454/1682 0.1(0.0,30.5) 99.86%(<0.001)
0
25
50
75
100
Incidence %

PRISMA 2009 Checklist

Section/topic	#	Checklist item	Reported on page #
**TITLE**			
Title	1	Identify the report as a systematic review, meta-analysis, or both.	1/title
**ABSTRACT**			
Structured summary	2	Provide a structured summary including, as applicable: background; objectives; data sources; study eligibility criteria, participants, and interventions; study appraisal and synthesis methods; results; limitations; conclusions and implications of key findings; systematic review registration number.	1/abstract
**INTRODUCTION**			
Rationale	3	Describe the rationale for the review in the context of what is already known.	4/introduction
Objectives	4	Provide an explicit statement of questions being addressed with reference to participants, interventions, comparisons, outcomes, and study design (PICOS).	4/introduction
**METHODS**			
Protocol and registration	5	Indicate if a review protocol exists, if and where it can be accessed (e.g., Web address), and, if available, provide registration information including registration number.	5/methods
Eligibility criteria	6	Specify study characteristics (e.g., PICOS, length of follow-up) and report characteristics (e.g., years considered, language, publication status) used as criteria for eligibility, giving rationale.	5/methods
Information sources	7	Describe all information sources (e.g., databases with dates of coverage, contact with study authors to identify additional studies) in the search and date last searched.	5/methods
Search	8	Present full electronic search strategy for at least one database, including any limits used, such that it could be repeated.	5/methods
Study selection	9	State the process for selecting studies (i.e., screening, eligibility, included in systematic review, and, if applicable, included in the meta-analysis).	6/methods
Data collection process	10	Describe method of data extraction from reports (e.g., piloted forms, independently, in duplicate) and any processes for obtaining and confirming data from investigators.	6/methods
Data items	11	List and define all variables for which data were sought (e.g., PICOS, funding sources) and any assumptions and simplifications made.	6/methods
Risk of bias in individual studies	12	Describe methods used for assessing risk of bias of individual studies (including specification of whether this was done at the study or outcome level), and how this information is to be used in any data synthesis.	6/methods
Summary measures	13	State the principal summary measures (e.g., risk ratio, difference in means).	6/methods
Synthesis of results	14	Describe the methods of handling data and combining results of studies, if done, including measures of consistency (e.g., I^2) for each meta-analysis.	6/methods

PRISMA 2009 Checklist

Section/topic	#	Checklist item	Reported on page #
Risk of bias across studies	15	Specify any assessment of risk of bias that may affect the cumulative evidence (e.g., publication bias, selective reporting within studies).	6/methods
Additional analyses	16	Describe methods of additional analyses (e.g., sensitivity or subgroup analyses, meta-regression), if done, indicating which were pre-specified.	6/methods
**RESULTS**			
Study selection	17	Give numbers of studies screened, assessed for eligibility, and included in the review, with reasons for exclusions at each stage, ideally with a flow diagram.	7/results
Study characteristics	18	For each study, present characteristics for which data were extracted (e.g., study size, PICOS, follow-up period) and provide the citations.	7/results
Risk of bias within studies	19	Present data on risk of bias of each study and, if available, any outcome level assessment (see item 12).	8/results
Results of individual studies	20	For all outcomes considered (benefits or harms), present, for each study: (a) simple summary data for each intervention group (b) effect estimates and confidence intervals, ideally with a forest plot.	9/results
Synthesis of results	21	Present results of each meta-analysis done, including confidence intervals and measures of consistency.	9/results
Risk of bias across studies	22	Present results of any assessment of risk of bias across studies (see Item 15).	10/results
Additional analysis	23	Give results of additional analyses, if done (e.g., sensitivity or subgroup analyses, meta-regression [see Item 16]).	11/results
**DISCUSSION**			
Summary of evidence	24	Summarize the main findings including the strength of evidence for each main outcome; consider their relevance to key groups (e.g., healthcare providers, users, and policy makers).	11/discussion
Limitations	25	Discuss limitations at study and outcome level (e.g., risk of bias), and at review-level (e.g., incomplete retrieval of identified research, reporting bias).	15/discussion
Conclusions	26	Provide a general interpretation of the results in the context of other evidence, and implications for future research.	16/discussion
**FUNDING**			
Funding	27	Describe sources of funding for the systematic review and other support (e.g., supply of data); role of funders for the systematic review.	17/funding

From: Moher D, Liberati A, Tetzlaff J, Altman DG, The PRISMA Group (2009). Preferred Reporting Items for Systematic Reviews and Meta-Analyses: The PRISMA Statement. PLoS Med 6(6): e1000097. doi:10.1371/journal.pmed1000097

For more information, visit: **www.prisma-statement.org**.
