# Supplementary figures and images for: Treatment‐related adverse events of antibody‐drug conjugates in clinical trials: A systematic review and meta‐analysis
Source: Cancer Innov. 2023 Oct 15;2(5):346–75. doi: 10.1002/cai2.97 (PMC10686142; doi:10.1002/cai2.97)

A

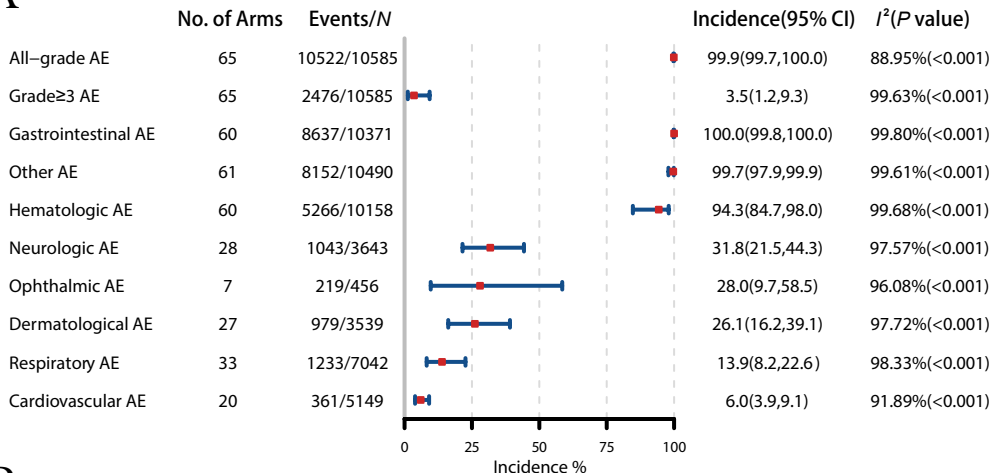

B

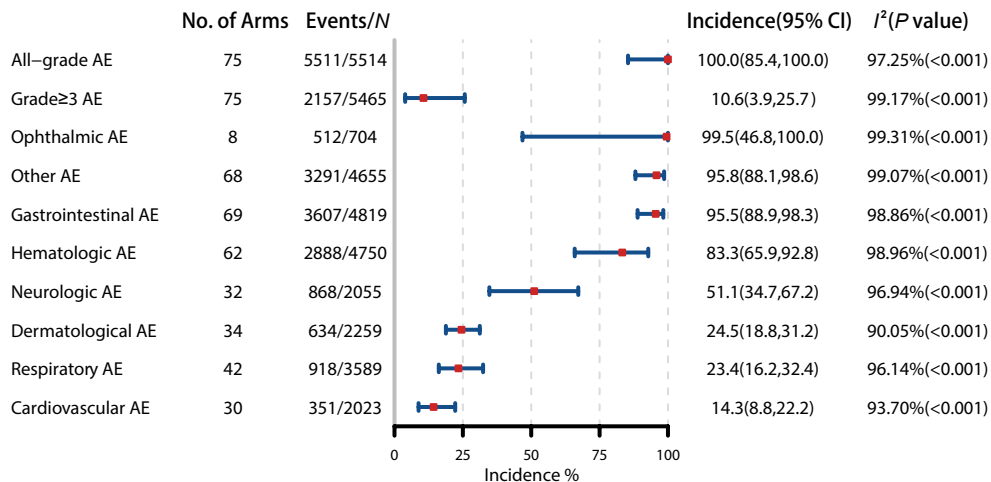

Supplement: Supplementary file 6 — eFigure 2. Overall incidence of adverse event according to cancer type. (A) Incidence of adverse events of all grades in patients with solid tumors and hematologic malignancies; (B) incidence of adverse events of grade ≥3 in patients with solid tumors and hematologic malignancies. [file CAI2-2-346-s003.pdf]

A

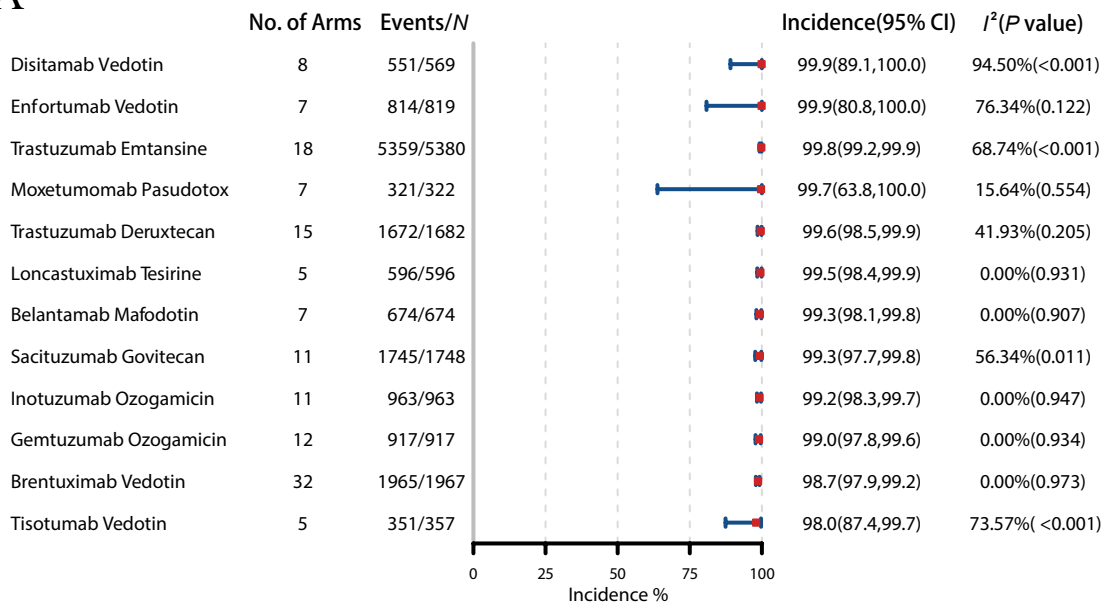

B

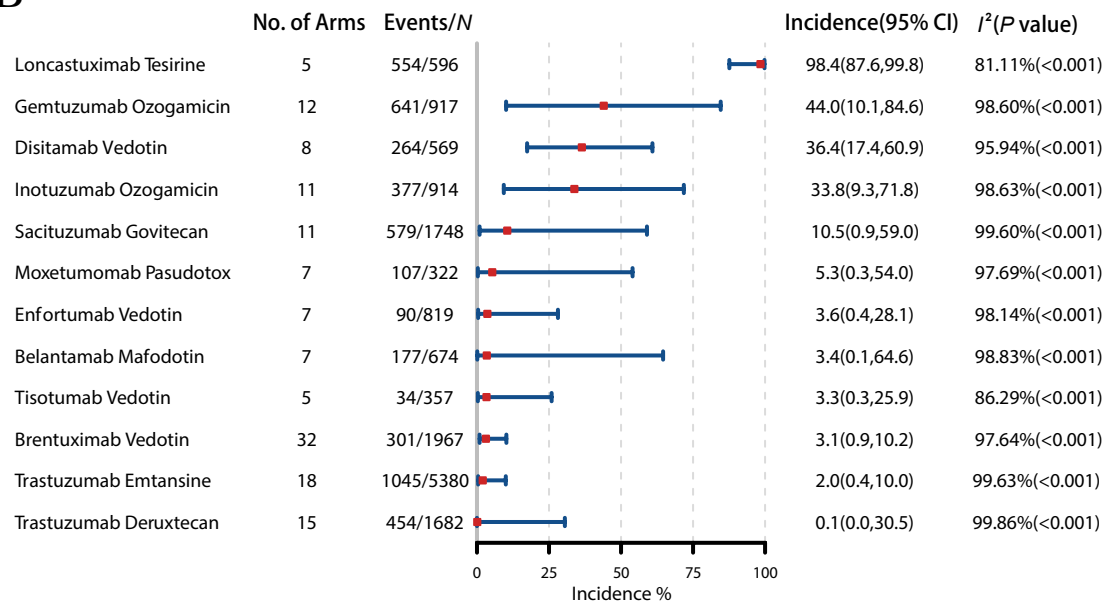

Supplement: Supplementary file 7 — eFigure 3. Overall incidence of adverse events based on ADC type. (A) Incidence of all grade adverse events based on ADC type; (B) incidence of grade ≥3 adverse events based on ADC type. [file CAI2-2-346-s001.pdf]

A

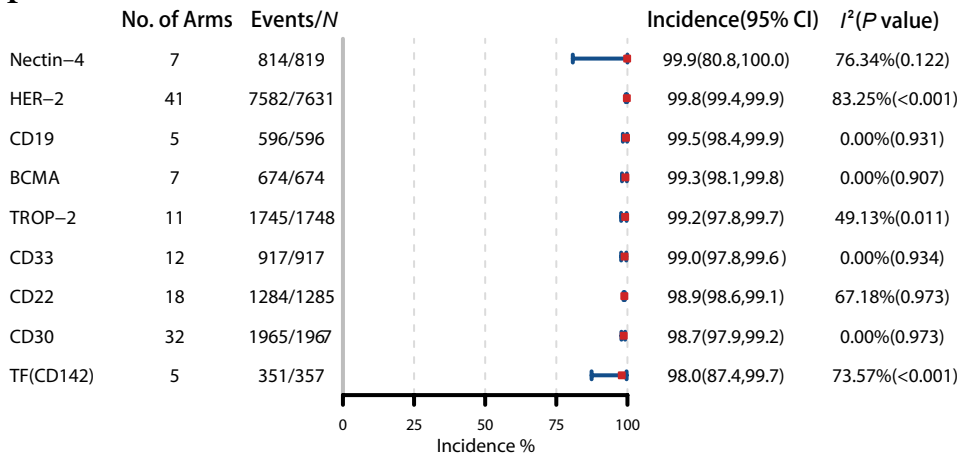

B

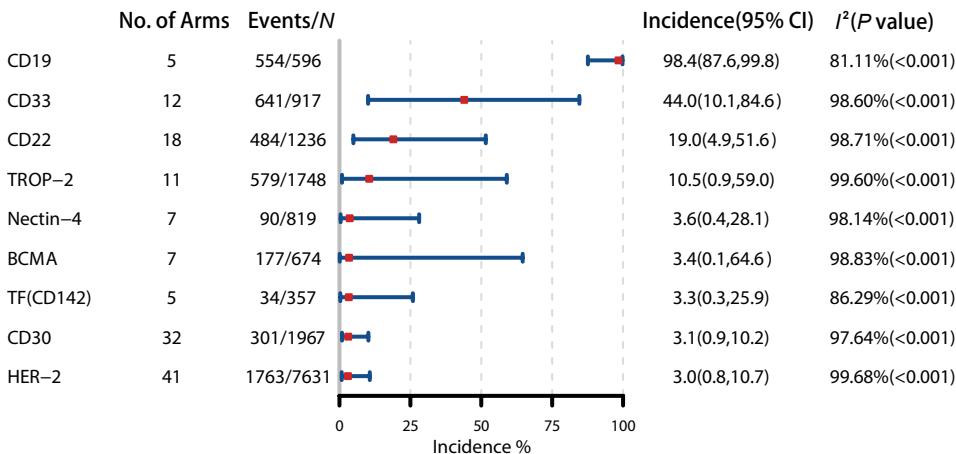

Supplement: Supplementary file 8 — eFigure 4. Overall incidence of adverse events based on antibody. (A) Incidence of all grade adverse events based on antibody; (B) incidence of grade ≥3 adverse events based on antibody. [file CAI2-2-346-s008.pdf]

A

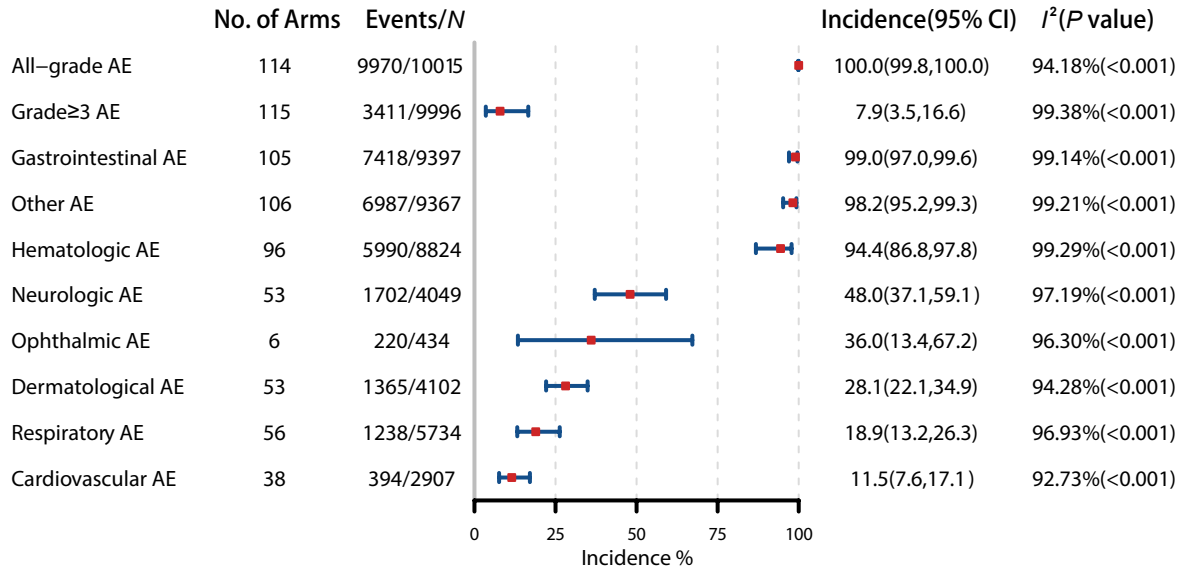

B

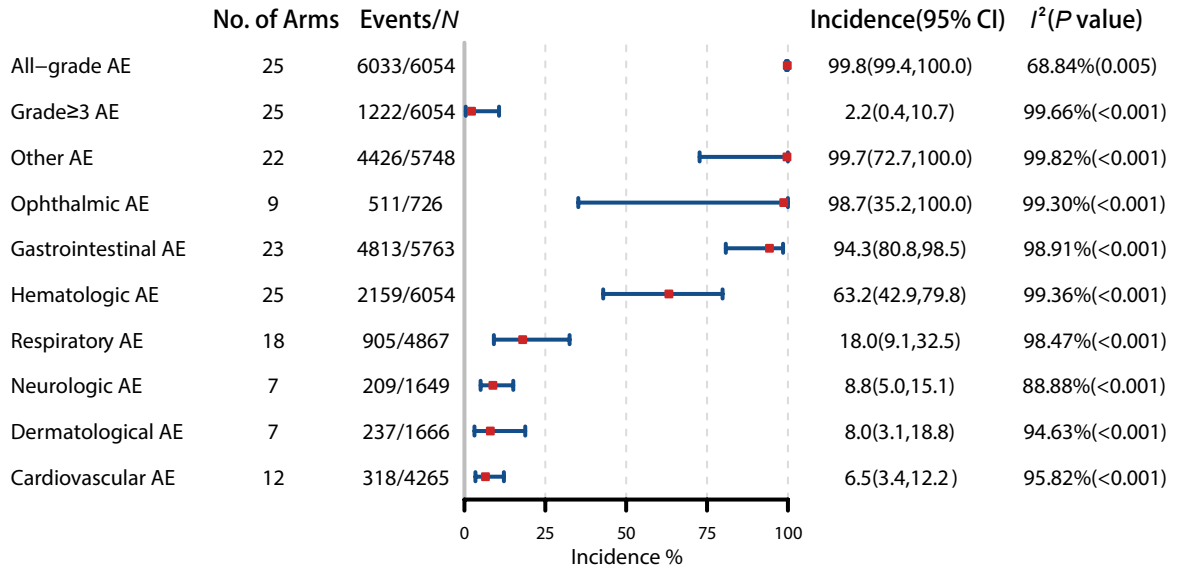

Supplement: Supplementary file 9 — eFigure 5. Overall incidence of adverse events based on linker. (A) Incidence of all grades of adverse events based on linker; (B) incidence of grade ≥3 adverse events based on linker. [file CAI2-2-346-s009.pdf]

A

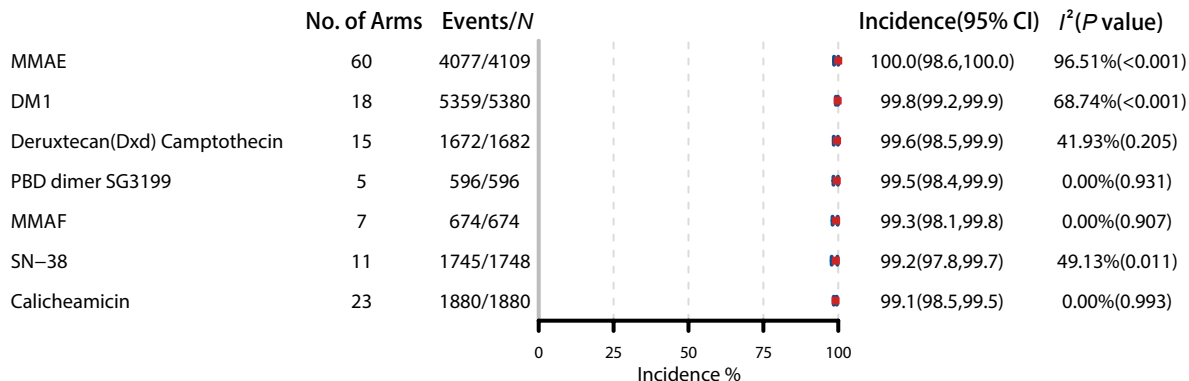

B

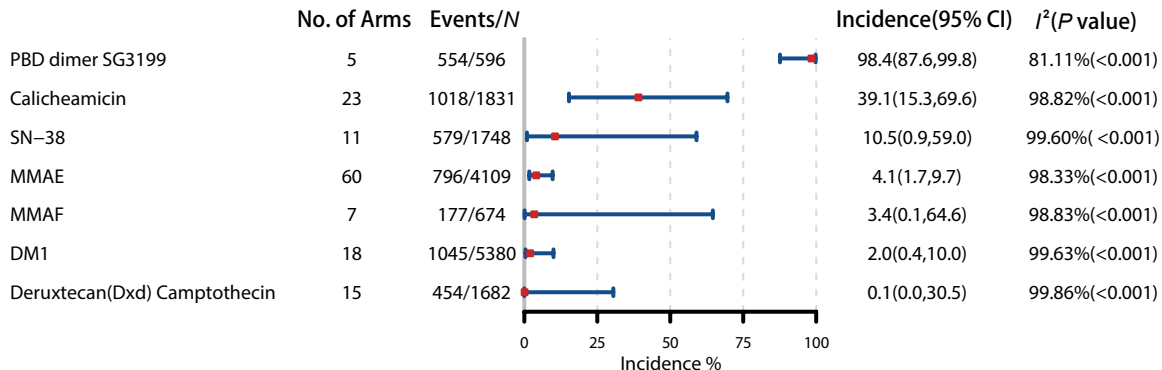

Supplement: Supplementary file 10 — eFigure 6. Overall incidence of adverse events based on payload. (A) Incidence of all‐grade adverse events based on payload; (B) incidence of grade ≥3 adverse events based on payload. [file CAI2-2-346-s007.pdf]
